# Metal‐Support Interaction Triggered Electronic Reconstruction in Pd/CoN‐Co_4_N Catalysts for Optimizing MEK Oxidation and Poisoning Resistance

**DOI:** 10.1002/advs.75899

**Published:** 2026-06-02

**Authors:** Yadi Wang, Chaoqian Ai, Cai Wang, Zeyu Jiang, Jialei Wan, Jingjing Wang, Xiangbo Feng, Chi He

**Affiliations:** ^1^ Shaanxi Key Laboratory of Liquid Crystal Polymer Intelligent Display Technological Institute of Materials & Energy Science (TIMES) Xijing University Xi'an P. R. China; ^2^ State Key Laboratory of Multiphase Flow in Power Engineering School of Energy and Power Engineering Xi'an Jiaotong University Xi'an Shaanxi P. R. China; ^3^ College of Engineering Xi'an International University Xi'an P. R. China; ^4^ National Engineering Laboratory for VOCs Pollution Control Material & Technology University of Chinese Academy of Sciences Beijing P. R. China

**Keywords:** electron transfer, electronic metal‐support interaction, Lewis acid sites, methyl ethyl ketone oxidation, poisoning resistance mechanism

## Abstract

The development of noble metal catalysts integrating high activity with strong poisoning resistance remains highly desirable yet challenging. Herein, the metallic electron‐donating property of a cobalt nitride support is leveraged to enhance the electron metal‐support interaction (EMSI) with Pd, concurrently improving the activity and poisoning resistance of Pd/CoN‐Co_4_N in methyl ethyl ketone (MEK) oxidation. Over which, 90% of MEK is oxidized at *ca*. 172 °C by Pd/CoN‐Co_4_N, versus only 48% conversion over Pd/Co_3_O_4_. Crucially, Pd/CoN‐Co_4_N exhibits exceptional resistance to H_2_S and 1,2‐dichloroethane poisoning, with performance showing almost no degradation upon H_2_S exposure, which completely deactivates Pd/Co_3_O_4_. The strong EMSI induces a profound electronic redistribution, facilitating electron transfer from the support to Pd and generating electron‐rich Pd^0^ *in situ*. This modulates the Pd *d*‐band center toward the Fermi level, enhancing MEK and O_2_ adsorption/activation and promoting deep oxidation of carboxylate intermediates. Furthermore, the enhanced EMSI creates abundant Lewis acid sites by modulating the charge density of Co atoms. These sites serve as sacrificial adsorption sites for electron‐rich poison molecules, thus shielding the Pd sites and facilitating rapid contaminant desorption. This work provides atomic‐level insight into EMSI on non‐oxide supports and proposes a design principle for robust catalysts toward environmental purification.

## Graphical abstract

The strengthened EMSI in Pd/CoN‐Co_4_N induces electronic rearrangement, generating abundant Pd^0^ species *in situ*, modulating the Pd *d*‐band center to enhance O_2_ adsorption and MEK activation. Concurrently, it creates numerous Lewis acid sites that preferentially adsorb poisons and shield the active sites. This multifaceted mechanism of the EMSI enables the concurrent high activity and poisoning resistance.

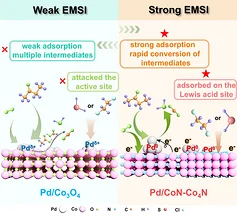

## 1 Introduction

The extensive emission of oxygenated volatile organic compounds (OVOCs) poses severe threats to the environment and human health, making their efficient catalytic purification a research priority in atmospheric pollution control [1, 2, 3]. Methyl ethyl ketone (MEK), a typical industrial OVOCs, exhibits exceptional molecular stability due to its synergistic *σ*‐conjugation system involving *sp* ^2^‐hybridized C═O and *sp* ^3^‐hybridized *σ* bonds, which hinders its low‐temperature activation [4]. Moreover, the performance of catalysts can be severely affected by the coexistence of sulfur‐ and chlorine‐containing impurities. Sulfur‐containing pollutants can be further oxidized to highly reactive SO_3_ intermediates, binding with surface hydroxyl/metal sites to generate sulfate overlayers [5, 6]. Chlorine‐containing pollutants compete with target molecules for adsorption *via* polar C─Cl bonds and form thermodynamically stable M─Cl coordination structures over active sites through nucleophilic substitution [3, 7]. The chemisorption of poison molecules not only blocks active sites but perturbs the electronic structure of adjacent metal atoms, disrupting their catalytic function and causing severe deactivation [8]. Therefore, developing catalysts with superb oxidation ability and poisoning resistance is crucial for OVOCs purification.

The purposeful modulation of electronic metal‐support interactions (EMSI) in catalysts to optimize the electronic structure of active centers is an effective strategy to enhance catalytic performance [9, 10]. Beyond stabilizing noble metal atoms through thermodynamically favorable metal‐support bonds, a robust EMSI is pivotal in enhancing catalyst poisoning resistance *via* electronic and bifunctional effects [11, 12]. Notably, the orbital rehybridization and charge transfer at the interface induce EMSI and alter the metal *d*‐band center. This electronic structure modification serves a dual purpose: it optimizes the adsorption of reaction intermediates and concurrently weakens the binding strength of catalyst poisons, thereby reducing their irreversible adsorption [13, 14]. Meanwhile, the strong interaction can modulate the electronic structure of the support, facilitating the generation of reactive species at the metal‐support interface. These species can act as scavengers to continuously oxidize and remove poisoning adsorbates from the active metal surface, thus restoring the activity through a bifunctional mechanism [15]. Nevertheless, precisely designing EMSI strength to maintain deep oxidation activity for OVOCs while improving poisoning resistance remains a critical scientific challenge that urgently needs to be addressed in this field.

A key obstacle to the durable use of Pd‐based catalysts in industrial settings is their limited poisoning resistance [16]. Selecting appropriate supports to construct EMSI can induce charge redistribution, further enabling adjustments to the local electronic environment of Pd. In recent years, transition metal nitrides (TMNs) have attracted widespread attention as novel support materials [17, 18]. As a class of interstitial compounds between pure metals and metal oxides, TMNs present exceptional catalytic performance across a lot of fields due to their high chemical reactivity, unique electronic structures, and corrosion resistance [19]. Nitrogen atoms induce lattice expansion in their metallic frameworks, influencing electron distribution and thereby modifying the electron‐donating capabilities of TMNs [20, 21]. Nevertheless, the complex interfacial electron transfer mechanisms between noble metal atoms and non‐oxide supports remain to be further elucidated. A deeper understanding of these mechanisms is key to synergistically optimizing both activity and stability at the molecular level.

Herein, we devised a gradient nitridation strategy to engineer the EMSI in Pd catalysts supported on cobalt oxides/nitrides. The optimized Pd/CoN‐Co_4_N catalyst exhibits remarkable resistance to poisoning while maintaining superior MEK oxidation activity. It elucidates that the intensified EMSI induces a dual mechanism: it drives electron donation to Pd, generating electron‐rich Pd^0^ sites that optimize O_2_ activation and MEK oxidation, while simultaneously creating electron‐deficient Lewis acid sites on the nitride support. These acid sites preferentially adsorb H_2_S and 1,2‐dichloroethane with lone‐pair electrons, thereby protecting Pd^0^ and maintaining stable catalytic performance during reactions. This work highlights the regulation of EMSI *via* support nitridation as an effective strategy for designing high‐performance and poison‐resistant catalysts toward OVOCs purification.

## 2 Results and Discussion

### 2.1 Structural Properties

**FIGURE 1:**
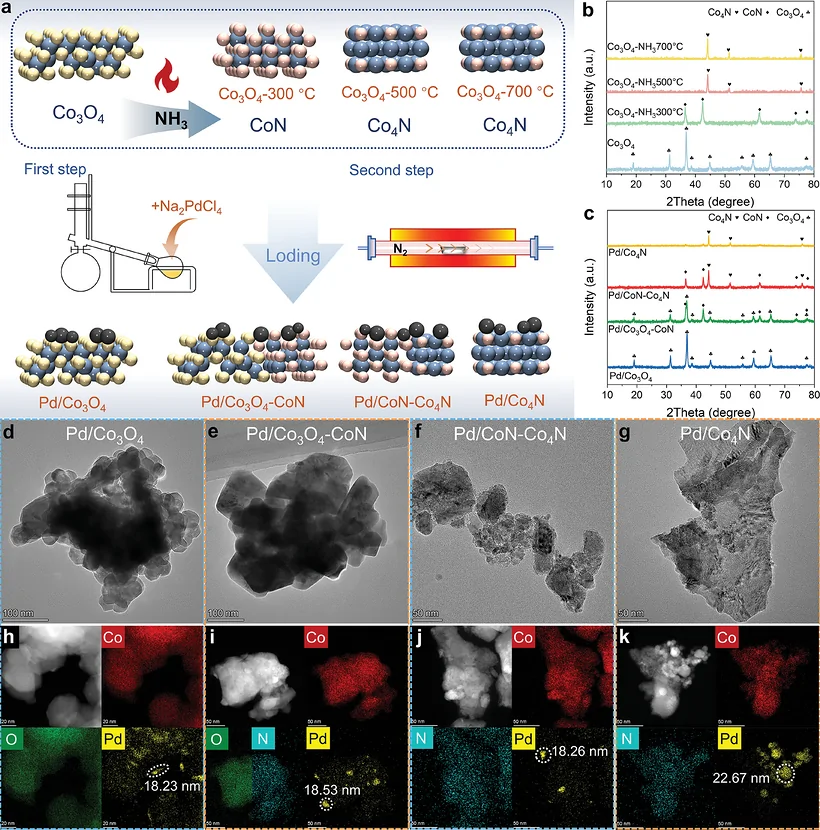
(a) Schematic illustration of supported‐Pd catalysts; (b, c) XRD patterns of supports and supported‐Pd catalysts; (d–g) TEM and (h–k) EDS mapping images of supported‐Pd catalysts.

The synthesis processes of catalysts are illustrated in Figure 1a. Cobalt nitride supports were synthesized *via* a nitridation process, and Pd nanoparticles were subsequently loaded using the rotary evaporation method. The evolution of the crystal structure of the cobalt‐based supports at various nitridation temperatures, along with their corresponding Pd‐loaded catalysts, was characterized by X‐ray diffraction (XRD). As shown in Figure 1b,c, the crystal structure of Pd/Co_3_O_4_ is consistent with that of Co_3_O_4_ support (PDF #74‐1656). However, significant structural changes occur in the supports after nitridation at 300 °C and 500 °C, both before and after Pd loading. Specifically, when the support was nitrided at 300 °C, the XRD pattern reveals complete transformation of Co_3_O_4_ into a single CoN phase (PDF #83‐0831), while the support nitrided at 500 °C is converted to a pure Co_4_N phase (PDF #41‐0943). Notably, the supports undergo remarkable structural reorganization after Pd loading. The NH_3_‐300 °C support (calcination of Co_3_O_4_ at 300 °C under NH_3_) forms a coexisting structure of Co_3_O_4_ and CoN (Pd/Co_3_O_4_‐CoN), whereas the NH_3_‐500 °C support (calcination of Co_3_O_4_ at 500 °C under NH_3_) evolves into a mixed phase of CoN and Co_4_N (Pd/CoN‐Co_4_N). Additionally, the NH_3_‐700 °C support (calcination of Co_3_O_4_ at 700 °C under NH_3_) largely maintains the single‐phase Co_4_N structure after Pd loading. No characteristic diffraction peaks associated with Pd nanoparticles were detected in all samples, confirming their well‐dispersed state on the catalyst surface.

The morphology of catalysts was characterized by transmission electron microscopy (TEM) and scanning electronic microscopy (SEM) (Figure 1d–g; Figure S1). As the nitridation temperature progressively increases (from 300 °C to 700 °C), the catalyst morphology gradually evolves into large bulk particles. Compared to the low‐temperature calcined (300 °C) sample, the Pd/CoN‐Co_4_N and Pd/Co_4_N catalysts display rougher surfaces with significant sintering and increased particle sizes. The morphology transitions from polygonal nanoparticles with distinct edges to 2D nanoplatelets. This morphological evolution is primarily attributed to the lattice reconstruction and particle agglomeration effects during high‐temperature nitridation [22].

Based on the results of energy dispersive spectroscopy (EDS) mapping (Figure 1h–k), it is evident that, except for localized enrichment of Pd species, all other elements remain uniformly dispersed on the catalyst surface. Notably, the particle size of Pd in the Pd/Co_4_N catalyst increases significantly. Figure S2 displays high‐resolution transmission electron microscopy (HR‐TEM) images of all catalysts. In Pd/Co_3_O_4_, characteristic lattice fringes with a spacing of 0.465 nm can be observed, corresponding to the (111) plane of Co_3_O_4_. The Pd/Co_3_O_4_‐CoN catalyst exhibits a dual crystal lattice structure, i.e., 0.461 nm, attributable to the (111) plane of Co_3_O_4_ and 0.238 nm, corresponding to the (111) plane of CoN. The lattice spacings of 0.247 and 0.211 nm, corresponding to the (111) planes of CoN and Co_4_N, respectively, are observed in the Pd/CoN‐Co_4_N catalyst. Meanwhile, the Pd/Co_4_N catalyst displays regular lattice fringes of 0.213 nm, unambiguously assigned to the (111) plane of Co_4_N. The observations demonstrate that the same type supports maintain consistent crystallographic parameters across different catalysts, thereby providing experimental evidence for the construction of precise density functional theory (DFT) calculation models (Figure S3). To further quantify the interfacial stability, the formation energy (*E*_f_) of the relevant systems was systematically calculated (Figure S4). The formation energy of CoN‐Co_4_N heterojunction is −4.27 eV. This negative value demonstrates that the formation of a tight heterojunction interface between CoN and Co_4_N is thermodynamically more stable than phase separation. The *E*_f_ of Pd/CoN‐Co_4_N is −3.98 eV, while that of Pd/Co_3_O_4_ is −2.58 eV. This indicates that loading Pd nanoparticles onto the stable CoN‐Co_4_N heterojunction results in a system with even superb overall stability than the conventional Pd/Co_3_O_4_ catalyst. The EDS mapping results confirm that the actual size distribution of Pd nanoparticles is significantly correlated with the agglomeration state of the support particles. The structural evolution of the cobalt nitride support leads to the reduced dispersion of active components. Inductively coupled plasma optical emission spectrometry (ICP‐OES) determines that the actual Pd content in all catalysts remains stable at approximately 0.45 wt.%, closely matching with the theoretical content (Table S1).

The chemical structures of the catalysts were further analyzed using Fourier transform infrared spectroscopy (FT‐IR) and Raman spectroscopy. As shown in Figure S5, Pd/Co_3_O_4_ and Pd/Co_3_O_4_‐CoN catalysts possess two characteristic absorption peaks at 571 and 665 cm^−1^, which are assigned to the stretching vibrations of octahedrally coordinated Co^3+^‐O and Co^2+^‐O structures, respectively [23, 24]. It confirms the existence of mixed Co^2+^ and Co^3+^ species on catalyst surfaces. With the introduction of N species, the intensity of these peaks significantly weakens. In particular, no Co^3+^‐O or Co^2+^‐O structures are observed in Pd/CoN‐Co_4_N and Pd/Co_4_N, indicating that these catalysts contain virtually no Co sites coordinated with O, and thus only Co─N bonds exist. As illustrated in Figure S6, the Raman spectra of all catalysts display four characteristic peaks. The peaks at 692, 483, 522, and 194 cm^−1^ correspond to A_1g_, E_g_, and two F_2g_ vibrational modes, respectively, which are characteristic of Co─O and Co─N bonds in the cobalt‐based compounds [25, 26]. Notably, the A_1g_ peak shows a red shift from 691 to 686 cm^−1^ with increasing nitridation degree, which is attributed to the expansion of metal‐metal lattice parameters [27, 28]. This phenomenon can be attributed to the electron cloud rearrangement around Co during the structural transformation from cobalt oxide to nitride, altering bond lengths and electronic environments [29, 30]. Additionally, as revealed by *in situ* DRIFTS of CO adsorption (CO‐DRIFTS) (Figure S7), two diffuse reflectance peaks at approximately 2172 and 2116 cm^−1^ are assigned to the gaseous CO (CO_gas_) and linearly adsorbed CO on Pd nanoparticles in all samples, respectively [31, 32].

### 2.2 Electronic Metal‐Support Interaction Identification

**FIGURE 2:**
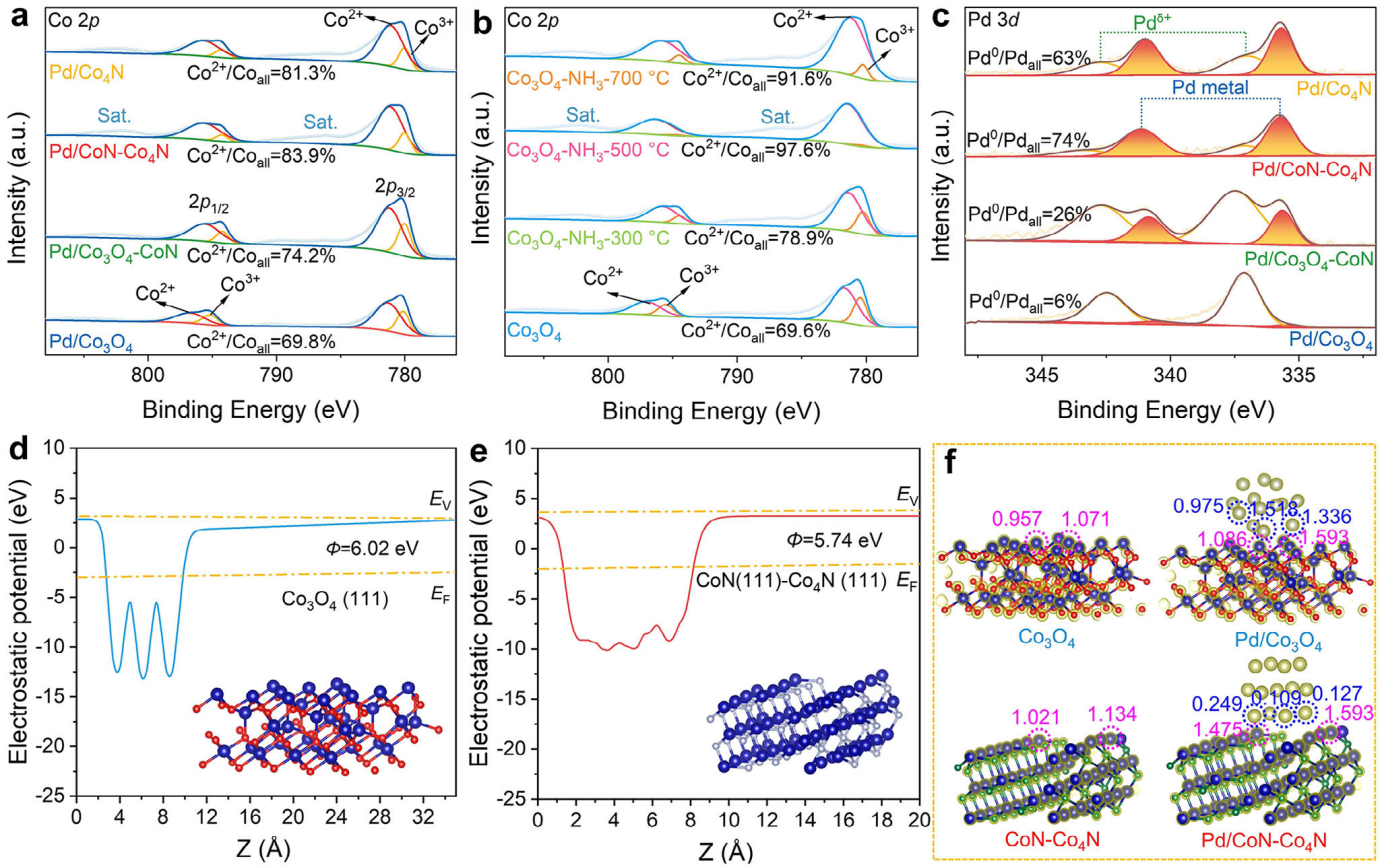
(a, b) Co 2*p* XPS spectra of supported‐Pd catalysts and the supports with diverse nitriding temperatures; (c) Pd 3*d* XPS spectra of supported‐Pd catalysts. Work function of (d) Co_3_O_4_(111), (e) CoN(111)‐Co_4_N(111) from DFT studies. (f) Mulliken charge analysis of the metal sites for Co_3_O_4_, Pd/Co_3_O_4_, CoN‐Co_4_N, and Pd/CoN‐Co_4_N.

X‐ray photoelectron spectroscopy (XPS) was applied to determine the electronic states of Co and Pd species. As shown in Figure 2a, the high‐resolution Co 2*p* XPS spectra are composed of two typical spin‐orbit doublets (Co 2*p*_3/2_ and Co 2*p*_1/2_) and satellite peaks (denoted as “Sat”). The Co 2*p*_3/2_ region can be further deconvoluted into two characteristic peaks at binding energies of 780.4 and 781.7 eV, corresponding to Co^3+^ and Co^2+^ species, respectively [33, 34]. All catalysts exhibit mixed Co valence states. Deconvolution results reveal that Pd/CoN‐Co_4_N has the highest proportion of tetrahedrally coordinated Co^2+^/Co_all_ (83.9%). The significant difference in Co^2+^ content suggests varying degrees of electron transfer within prepared catalysts.

To further investigate the interaction between metal and support, the XPS of the support was tested (Figure 2b). Given that nitrogen has a higher electronegativity than oxygen, its incorporation into bulk cobalt oxide tunes the electronic distribution of surrounding metal atoms, thereby modifying the valence states of Co atoms. Moreover, the results indicate that the proportion of Co^2+^ remains nearly unchanged (only in Co_3_O_4_ support) relative to that in Pd/Co_3_O_4_. Combined with Pd‐loaded Co 2*p* XPS results, the nitrided support exhibits enhanced Co^2+^ proportion modulation after Pd loading. Specifically, the Co^2+^/Co_all_ in Pd/Co_3_O_4_‐CoN decreases by 5.7% compared to the original nitrided support of Co_3_O_4_‐NH_3_ 500 °C. Compared to their original nitrided samples, the proportion decreases by 10.3% over the Pd/Co_4_N catalyst, while Pd/CoN‐Co_4_N shows the largest decrease in Co^2+^ proportion. Although the Co_4_N support derived from Co_3_O_4_ nitrided at 700 °C shares the same Co_4_N phase as the prepared sample at 500 °C, its initial Co^2+^ content is significantly lower due to electronic structure alterations caused by high‐temperature sintering.

In parallel, the Pd 3*d* XPS spectra reveal that only the Pd/Co_3_O_4_ catalyst shows a single chemical state corresponding to oxidized Pd species, whereas the other samples display both Pd^δ+^ and Pd^0^ states, accompanied by a significant binding energy shift to a lower value (Figure 2c) [35, 36]. The relative valence distribution of Pd species changes significantly with the nitriding degree of the support. As nitrogen atoms were incorporated into the support, a high concentration of metallic Pd species was *in situ* formed on the catalyst surface after Pd loading. Notably, Pd/CoN‐Co_4_N exhibits the highest Pd^0^/Pd_all_ proportion (74%), followed by Pd/Co_4_N (63%) and Pd/Co_3_O_4_‐CoN (26%). It evidently demonstrates the EMSI between nitrided support and Pd nanoparticles. The facilitated EMSI, which is absent in the Pd/Co_3_O_4_ sample, promotes electron transfer from Co to Pd atoms and induces the *in situ* generation of Pd^0^ over prepared nitrided samples. These results indicate that nitrided supports lose partial Co^2+^ electrons upon Pd loading (Co^2+^ → Co^3+^). The lost electrons undergo directional migration to Pd atoms, inducing significant electron transfer. This mechanism is most pronounced in Pd/CoN‐Co_4_N, where maximal Co electron loss accompanies enhanced Pd° formation. The findings rationalize the structural reconstruction of support, as proved by XRD patterns (Figure 1c).

The work function (*Φ*) was calculated by evaluating the energy difference between the vacuum level (*E*_V_) and Fermi level (*E*_F_) via DFT calculations based on the electrostatic potentials of two representative supports (Co_3_O_4_ and CoN‐Co_4_N), which can further confirm the electron transfer ability of the support [37, 38]. As shown in Figure 2d,e, the work functions of the Co_3_O_4_ and CoN‐Co_4_N are 6.02 and 5.74 eV, respectively. The significantly reduced work function of the latter indicates weakened surface electron binding energy, leading to facilitated electron escape from the surface and enabling spontaneous electron transfer [39]. This property enables CoN‐Co_4_N to serve as an efficient electron donor, transferring electrons to the Pd active centers through the metal‐support interface.

To explore the charge transfer effects induced by noble metal loading, the Mulliken charge analysis was performed on Co and Pd sites in Co_3_O_4_ and CoN‐Co_4_N supports before and after Pd loading, as shown in Figure 2f. The calculated Mulliken charge density of Co sites in Co_3_O_4_ is 0.957, closely matching that of Pd/Co_3_O_4_. In contrast, the charge densities of Co at the CoN‐Co_4_N heterojunction are 1.021 and 1.134, respectively, which significantly increase to 1.475 and 1.593 in the Pd/CoN‐Co_4_N catalyst. The Mulliken charge density around Pd atoms at the Pd‐Co interface in Pd/CoN‐Co_4_N is much lower than that in Pd/Co_3_O_4_, indicating that nitrogen substituted during thermal treatment induces charge redistribution between Co sites and Pd species in Pd/CoN‐Co_4_N, with net electron transfer from Co to interfacial Pd atoms [40]. Meanwhile, the orbital‐projected density of states (PDOS, Figures S8 and S9) shows that the Pd‐*d* states exhibit stronger hybridization with the N‐*p* states of the CoN‐Co_4_N support near the Fermi level than with the O‐*p* states in Pd/Co_3_O_4_, suggesting a modified interfacial electronic coupling [41]. To quantify the interfacial bonding strength, we performed crystal orbital Hamiltonian population (COHP) analysis on the key interfacial bonds. As shown in Figures S10 and S11, the integrated COHP (‐ICOHP) value, a direct descriptor of bond strength, for the Pd─N bond at the Pd/CoN‐Co_4_N interface is calculated to be −3.212 eV. In stark contrast, the ‐ICOHP for the Pd─O bond at the Pd/Co_3_O_4_ interface is only −1.625 eV. The nearly two‐fold stronger Pd─N bond provides direct quantitative evidence for the significantly enhanced covalent interaction at the nitride‐supported interface, corroborating the stronger EMSI effect [42]. This enhanced covalent interaction at the interface facilitates electron transfer. Accordingly, Bader charge analysis confirms a more pronounced electron donation from the nitride support to the Pd nanoparticles in Pd/CoN‐Co_4_N. The differential charge density plot visually corroborates this intensified charge redistribution at the interface (Figure S12) [43, 44]. Collectively, these results conclusively demonstrate that the nitridation strategy successfully leads to a much stronger EMSI.

### 2.3 Effect of EMSI on Catalytic Performance

**FIGURE 3:**
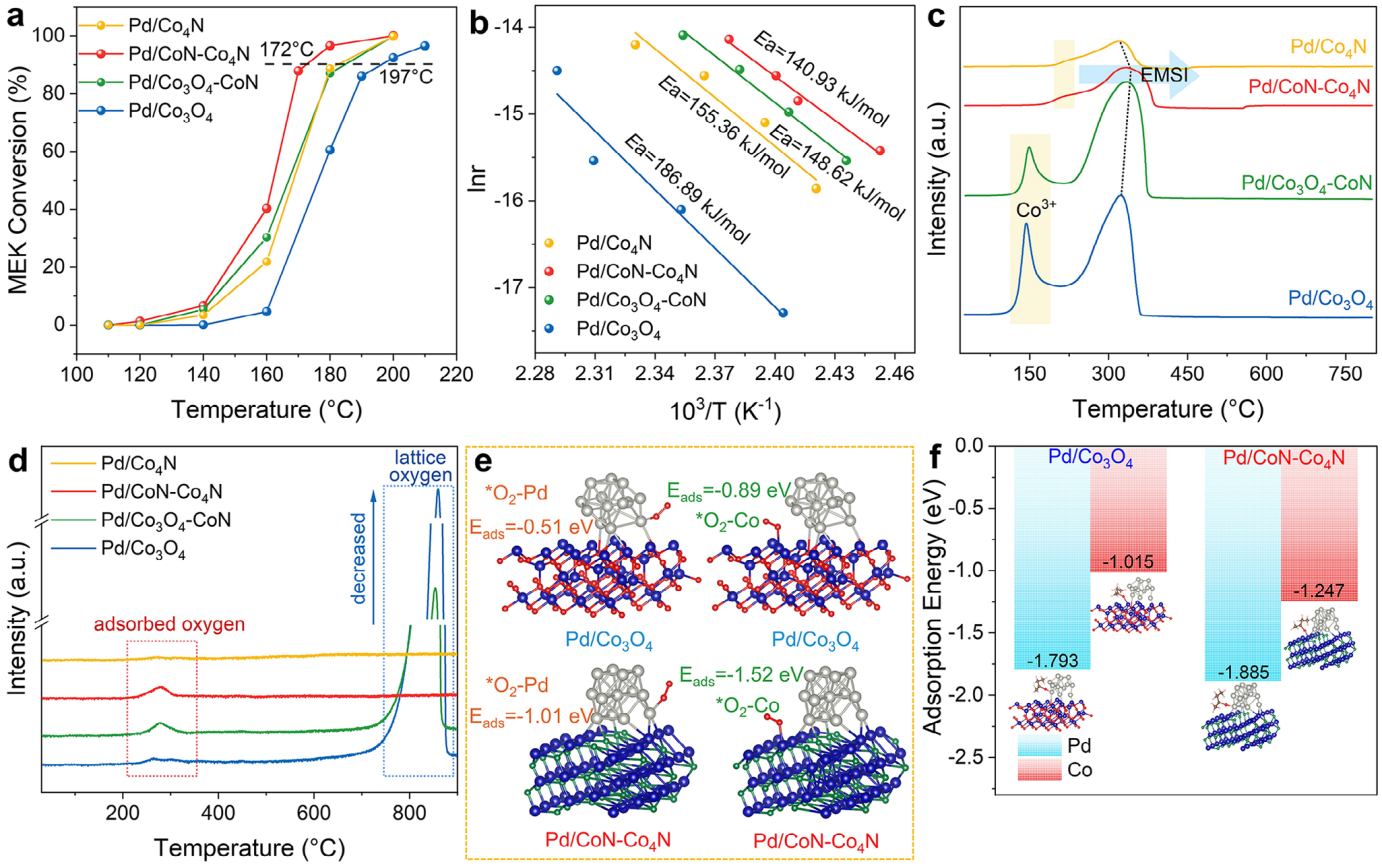
(a) Catalytic activity and (b) Arrhenius plots of MEK oxidaiton; (c) H_2_‐TPR and (d) O_2_‐TPD profile; (e) O_2_ adsorption energy on Pd and Co sites of Pd/Co_3_O_4_ and Pd/CoN‐Co_4_N; (f) MEK adsorption energy on interface Pd and Co sites of Pd/Co_3_O_4_ and Pd/CoN‐Co_4_N (color code: Co (blue), O (red), Pd (gray), N (green), C (brown), and H (pink)).

The catalytic performance of supported‐Pd catalysts was evaluated for methyl ethyl ketone oxidation. Figure 3a and Table S1 show that Pd/Co_3_O_4_ displays inferior catalytic activity among these catalysts, with the lowest activity at 160 °C. In contrast, all nitridation‐modified catalysts demonstrate significantly enhanced oxidation activity. Among them, Pd/Co_3_O_4_‐CoN and Pd/Co_4_N display nearly identical *T*_90_ (temperature for 90% conversion of MEK), while they show distinct differences in low‐temperature catalytic performance. The Pd/CoN‐Co_4_N catalyst, which features the CoN‐Co_4_N heterojunction structure, achieves the optimal catalytic performance for MEK (*T*_90_ of 172 °C). It is found that the activity gradient positively correlated with the strength of EMSI, where strong electron transfer effects notably improve interfacial catalytic reaction kinetics. The reduced activity of Pd/Co_4_N compared to Pd/CoN‐Co_4_N can be attributed to the support sintering and active site agglomeration. Moreover, in addition to minimizing sintering, the CoN‐Co_4_N heterojunction is deemed essential. The electric field at the interface between the two nitride phases presumably fosters a more conducive environment for electron donation than the separate phases do independently. This synergy amplifies the total EMSI strength, facilitating the enhanced performance of Pd/CoN‐Co_4_N. All the catalysts have well CO_2_ selectivity (Figures S13 and S14). Meanwhile, Pd/CoN‐Co_4_N exhibits remarkable activity compared to other catalysts (Table S2). The Arrhenius plots in Figure 3b demonstrate that the apparent activation energy (*E*_a_) of Pd/CoN‐Co_4_N (140.93 kJ/mol) is lower than that of other catalysts, revealing the advantage of Pd/CoN‐Co_4_N on activating reactants.

To evaluate the structural stability of Pd/CoN‐Co_4_N, long‐term stability and cycling stability tests were performed. As shown in Figure S15, the MEK conversion of the catalyst remains above 90% with no activity decay observed under a reaction temperature of 172 °C for 35 h of continuous operation. Furthermore, the cycling stability test results (Figure S16) reveal that Pd/CoN‐Co_4_N maintains performance close to its initial level even after three complete test cycles, demonstrating that its catalytic performance does not degrade due to prolonged reactions in air.

Hydrogen temperature‐programmed reduction (H_2_‐TPR) was performed to evaluate the reducibility of the catalysts, as shown in Figure 3c. Pd/Co_3_O_4_ and Pd/Co_3_O_4_‐CoN exhibit a prominent reduction peak in the range of 144 °C–149 °C, attributed to the reduction of Co_3_O_4_ to CoO [45]. By contrast, the Co^3+^ reduction peaks of Pd/CoN‐Co_4_N and Pd/Co_4_N shift to higher temperatures. All samples display a broad reduction peak at 322 °C–341 °C, corresponding to the deeper reduction of CoO to metallic Co. Notably, Pd/CoN‐Co_4_N exhibits the highest temperature for the second reduction peak among all catalysts. The elevated reduction temperature arises from the strong electronic coupling between Pd active sites and the cobalt nitride support, while the enhanced interaction simultaneously inhibits the deep reduction of Co^2+^ [46].

To gain deeper insights into the oxygen species characteristics in the catalysts, the O_2_ temperature‐programmed desorption (O_2_‐TPD) test was conducted, and the adsorption energy for oxygen was calculated. As shown in Figure 3d, the strong desorption peak above 700 °C in the high‐temperature region corresponds to the release of lattice oxygen species, which is only observed in Pd/Co_3_O_4_ and Pd/Co_3_O_4_‐CoN catalysts with oxygen‐containing supports [47]. In particular, the weak peak below 300 °C in the low‐temperature region is detected for Pd/Co_3_O_4_‐CoN and Pd/CoN‐Co_4_N catalysts, originating from chemisorbed oxygen species on the surface [48]. This demonstrates that nitrogen incorporation effectively enhances the oxygen adsorption capacity. The high electronegativity of N atoms withdraws electron density from adjacent Co sites, rendering them electron‐deficient. This enhances their affinity for the lone‐pair electrons of O_2_, thereby promoting chemisorption over mere physical adsorption. The increased concentration of surface chemisorbed oxygen significantly reduces the oxygen activation energy barriers, playing a crucial role in accelerating the kinetics of oxidation reactions. Furthermore, the adsorption energy of O_2_ adsorption sites was calculated for Pd/Co_3_O_4_ and Pd/CoN‐Co_4_N. In Figure 3e, the adsorption energies of O_2_ molecules on the Pd/Co_3_O_4_ support sites and Pd active sites are −0.89 and −0.51 eV, respectively, while those for Pd/CoN‐Co_4_N reduce markedly to −1.52 and −1.01 eV. These results confirm that oxygen activation in both catalysts preferentially occurs on the support surface, with Pd/CoN‐Co_4_N exhibiting lower oxygen adsorption energy. The energy barrier for O_2_ dissociation (^*^O_2_ → 2^*^O) was calculated (Figure S17). The barrier on Pd/CoN‐Co_4_N is only 0.32 eV, which is substantially lower than the 0.54 eV of Pd/Co_3_O_4_. This marked reduction demonstrates that the enhanced EMSI greatly facilitates the activation of molecular oxygen.

The MEK temperature‐programmed desorption (MEK‐TPD) (Figure S18) reveals that Pd/CoN‐Co_4_N exhibits the lowest chemisorption desorption temperature at 284 °C, enabling MEK to rapidly desorb to release more active sites. Meanwhile, the amount of physically adsorbed MEK is significantly higher than that of other catalysts, confirming the presence of abundant adsorption sites on the surface. It indicates that the Pd/CoN‐Co_4_N catalyst possesses excellent MEK adsorption‐activation synergy. In addition, DFT calculations were performed to investigate the adsorption energies of MEK on different sites (interfacial and bulk Pd, as well as Co sites) with different configurations over the Pd/Co_3_O_4_ and Pd/CoN‐Co_4_N catalysts (Figure 3f; Figure S19 and Table S3). On both catalysts, the interfacial Pd site exhibits the strongest adsorption for MEK *via* the carbonyl O atom, with adsorption energies of −1.793 and −1.885 eV, respectively. Meanwhile, adsorption through the carbonyl O atom is significantly stronger than that *via* the methyl C─H bond. This is attributed to the stronger orbital coupling between Pd and the carbonyl O atom, verifying that the most probable initial activation mode of MEK involves the bonding of the carbonyl oxygen to the active site. The lower adsorption energy on Pd/CoN‐Co_4_N indicates that the enhanced EMSI directly strengthens the catalyst's ability to adsorb MEK, thereby laying the foundation for the higher reaction activity. The more negative adsorption energies for both O_2_ and MEK on Pd/CoN‐Co_4_N indicate a facilitated initial activation of these key reactants. This optimized adsorption behavior effectively contributes to the lower apparent activation energy observed in kinetic measurements, synergistically enhancing the overall reaction rate. Moreover, the total density of states (TDOS) of Pd/Co_3_O_4_ and Pd/CoN‐Co_4_N exhibits non‐zero values at the Fermi level, indicating that electrons can move freely (Figure S20). Pd/CoN‐Co_4_N shows more pronounced variations in TDOS within this region, reflecting enhanced electronic activity. The *d*‐band center (*ε_d_*; the average position of the *d*‐orbital of the metal atom) of Pd/CoN‐Co_4_N is closer to the Fermi level (−0.5170 eV), which strengthens interactions with the anti‐bonding states of adsorbed molecules. This weakens the adsorption energy while improving adsorption ability, which is consistent with the more negative adsorption energies calculated, and ultimately contributes to the enhanced activation of these molecules. These findings demonstrate that the cobalt nitride support optimizes the electronic structure of Pd sites through strengthened EMSI, thereby enhancing MEK adsorption and activation efficiency.

The nitridation treatment induces a fundamental transformation of the support's structure, and the subsequent Pd loading facilitates a reconstruction of the catalyst's structure, leading directly to significant alterations in its electronic properties. In the Pd/CoN‐Co_4_N catalyst, this is manifested as a pronounced interfacial electron transfer, characterized by directional donation from the Co support to the Pd active centers. This strong EMSI effect facilitates the *in situ* reduction of Pd species to a high proportion of metallic Pd^0^ and optimizes their electronic structure, resulting in an upshift of the *d*‐band center closer to the Fermi level. The upshifted *d*‐band center strengthens the interaction with the anti‐bonding orbitals of MEK, thereby enhancing adsorption while concurrently optimizing the activation process. Furthermore, the energy barrier for oxygen adsorption and activation is significantly lowered in the Pd/CoN‐Co_4_N catalyst, which more readily facilitates the activation of molecular oxygen and the initiation of the reaction. These optimizations at the atomic and electronic levels, induced by the strong EMSI effect, do not rely on a single mechanism. Instead, they work by synergistically lowering the energy barriers across multiple steps along the reaction pathway. Such enhancements at the microscale promote faster interfacial reaction kinetics and reduce the *E*_a_, ultimately leading to a significant improvement in catalytic performance.

### 2.4 Catalytic Mechanism

**FIGURE 4:**
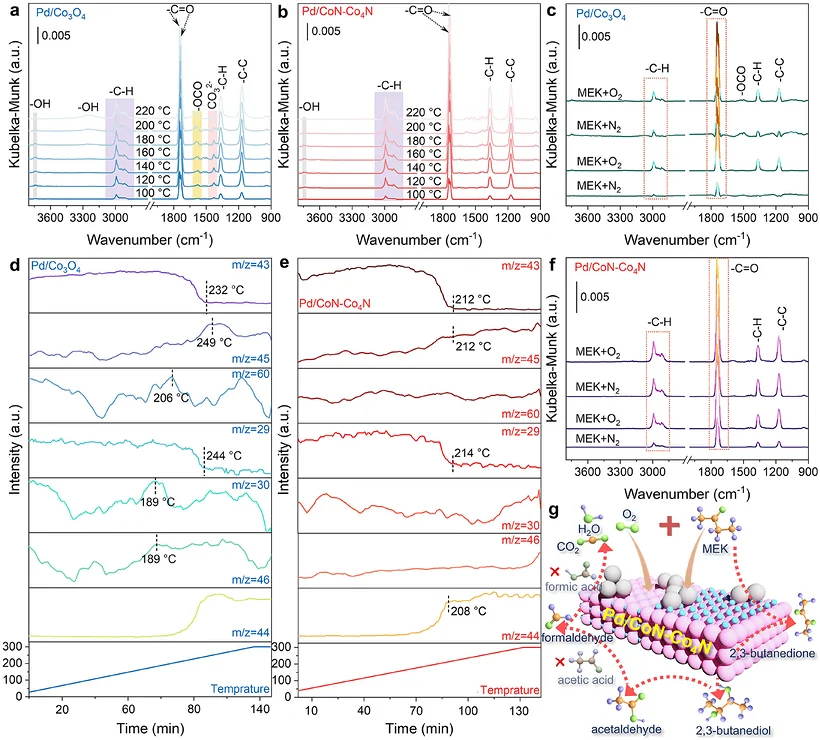
*In situ* DRIFTS of MEK oxidation (a, b) as a function of temperature from 100 °C to 220 °C and (c, f) under different reaction conditions at *T*_90_; (d, e) MEK‐TPSR profiles of Pd/Co_3_O_4_ and Pd/CoN‐Co_4_N; (g) Reaction mechanism of MEK oxidation over Pd/CoN‐Co_4_N catalyst (color code: Co (pink), N (blue), Pd (gray), O (green), C (orange), and H (violet)).

*In situ* diffuse reflectance infrared Fourier transform spectroscopy (*in situ* DRIFTS) was employed to investigate the adsorbed reactants and intermediates on the catalysts under varying temperatures. As shown in Figure 4a,b and Figures S21 and S22, all samples exhibit vibration peaks at 1366 and 2800–3080 cm^−1^, assigned to the bending and stretching vibrations of C─H bonds [49, 50]. A peak observed at 1744 cm^−1^ is attributed to the C═O vibration in MEK [51, 52, 53]. Additionally, the stretching vibration of the C─C bond appears at 1171 cm^−1^, confirming the continuous adsorption of MEK molecules during the reaction [54, 55]. The peak at 1732 cm^−1^ within the tested temperature range is assigned to the C═O stretching vibration in acetaldehyde or formaldehyde, indicating aldehydes as intermediate products for all samples [56]. The ─OH vibration at 3725 cm^−1^ suggests the generation of alcohol species during the reaction [57]. Furthermore, a band at 1425 cm^−1^ in Figure 4a and Figure S21 is attributed to surface‐accumulated monodentate carbonate species, while the peak at 1585 cm^−1^ arises from asymmetric stretching vibrations of carboxylate (─OCO) [58, 59]. These observations indicate that carboxylate intermediates and carbonate species tend to form and accumulate on Pd/Co_3_O_4_ and Pd/Co_3_O_4_‐CoN catalysts as temperature increases. In contrast, such peaks are absent in Pd/CoN‐Co_4_N with strong EMSI. Even at low temperatures, the absence of accumulated carboxylate or carbonate species on Pd/CoN‐Co_4_N indicates its superior deep oxidation ability. This phenomenon occurs because the rate of intermediate oxidation to CO_2_ exceeds its formation rate. Such enhanced performance is attributed to the optimized electronic structure of the Pd sites and their promoted oxygen activation ability. It is revealed that enhanced EMSI optimizes interfacial electron transfer pathways, effectively accelerating the deep oxidation of intermediates.

To further pinpoint the oxidation degradation pathways and intermediates of the catalysts, MEK temperature‐programmed surface reaction (MEK‐TPSR) tests were conducted. In Figure 4d,e and Figures S23 and S24, mass spectrometry analysis reveals that *m/z* = 43, assigned to acetyl ion fragments formed by butanedione cleavage into two carbonyl groups, exhibits significant signal intensity evolution across all catalysts, confirming that butanedione is initially generated during MEK oxidation. Pd/Co_3_O_4_ requires the highest temperature to fully convert butanedione, consistent with the trend of its CO_2_ formation peak (*m/z* = 44). In contrast, the Pd/CoN‐Co_4_N catalyst reaches the CO_2_ formation peak at 208 °C, indicating its superior deep oxidation ability for intermediates. Following complete butanedione conversion, fragments from butanediol cleavage into hydroxyl (─OH) and short carbon chains (*m/z* = 45) are detected on the catalysts. Notably, the *m/z* = 45 signal is significantly weaker on Pd/CoN‐Co_4_N, with only faint signals for acetaldehyde observed (*m/z* = 29), demonstrating its enhanced conversion efficiency. Importantly, formic acid (*m/z* = 46) and acetic acid (*m/z* = 60) signals are detected on Pd/Co_3_O_4_, and to a lesser extent on Pd/Co_3_O_4_‐CoN. The results combined with *in situ* DRIFTS reveal that MEK catalytic oxidation proceeds as follows: first, MEK reacts with surface‐adsorbed oxygen to form 2,3‐butanedione, which is then oxidized to 2,3‐butanediol, followed by conversion into acetaldehyde, acetic acid, formaldehyde, formic acid, and finally CO_2_ and H_2_O. The Pd/CoN‐Co_4_N restructures the interfacial reaction microenvironment through EMSI, and effectively blocks carboxylic acid intermediate formation pathways and directly promotes deep mineralization of aldehyde intermediates into final products (Figure 4g).

Subsequently, *in situ* DRIFTS were recorded at the *T*_90_ of each catalyst under different reaction conditions (MEK + O_2_/N_2_) to reveal the catalytic mechanism governing the MEK oxidation process on the catalyst. As shown in Figure 4c,f and Figures S25 and S26, when the reaction atmosphere is MEK + N_2_, adsorbed MEK molecules and vibrational signals attributed to aldehyde intermediates are detected on the surfaces of all catalysts. Pd/Co_3_O_4_ exhibits only weak MEK adsorption ability without O_2_. When the reaction atmosphere was switched from N_2_ to O_2_, the intensities of surface vibrational peaks for all catalysts were significantly increased, indicating that adsorbed oxygen dominates as an active oxygen species during the reaction. Comparative analysis shows that after switching the O_2_ back to N_2_, peak intensities notably decrease for Pd/Co_3_O_4_ and Pd/Co_3_O_4_‐CoN, further confirming their weaker adsorption capacity for O_2_ and MEK. The results strongly support that the primary active oxygen species for MEK oxidation on this series of catalysts is surface‐adsorbed O_2_ molecules, indicating that the reaction follows the Langmuir‐Hinshelwood mechanism model.

### 2.5 Poisoning Resistance

**FIGURE 5:**
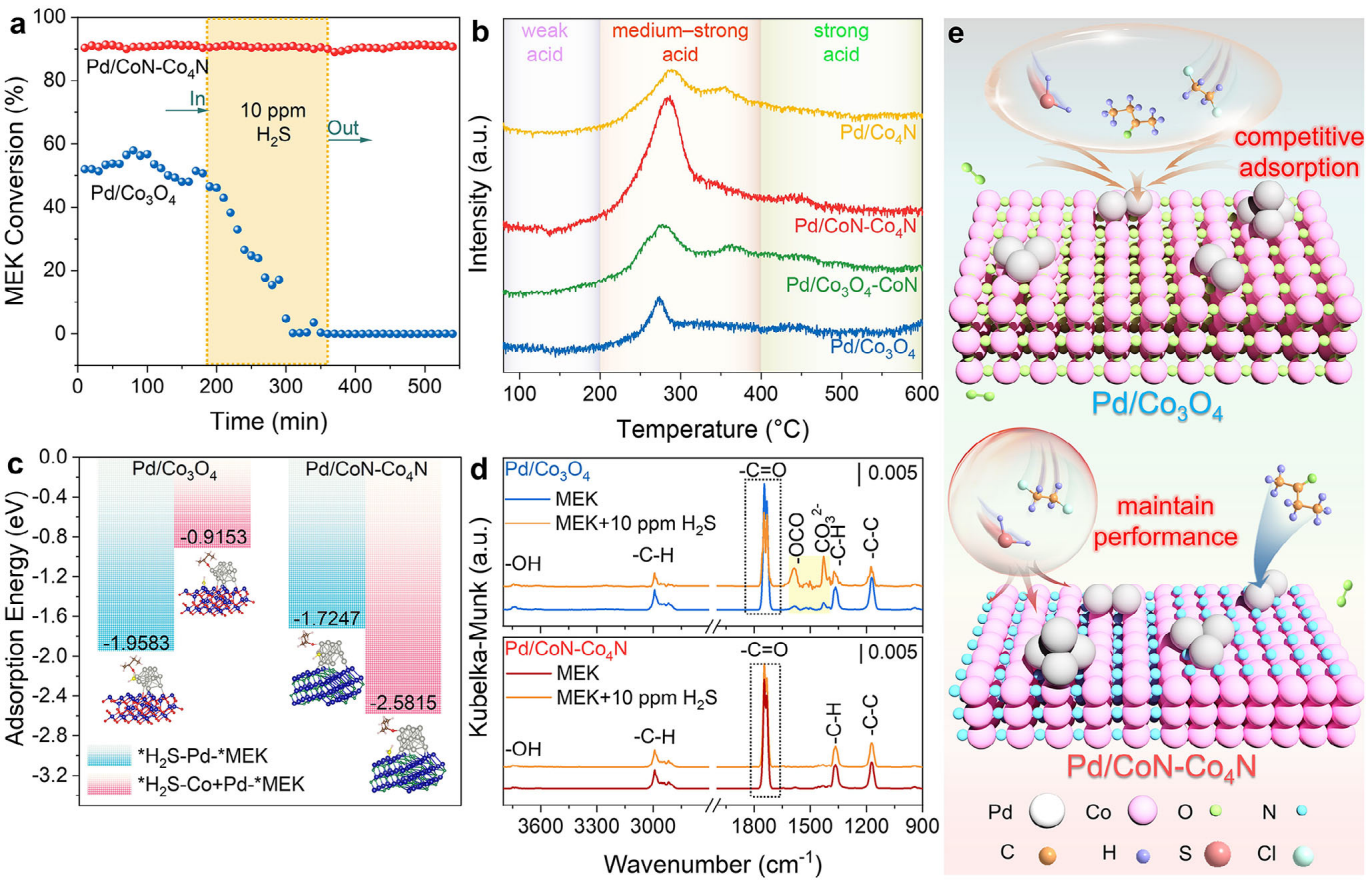
(a) H_2_S resistance test of Pd/Co_3_O_4_ and Pd/CoN‐Co_4_N for MEK oxidation; (b) MEK‐TPD of prepared catalysts; (c) The simulated configurations of H_2_S and MEK co‐adsorbed (color code: Co (blue), O (red), Pd (gray), N (green), C (brown), and H (pink)); (d) in situ DRIFTS of MEK oxidation with or without H_2_S on Pd/Co_3_O_4_ and Pd/CoN‐Co_4_N; (e) Mechanism diagram of poisoning resistance.

To evaluate the poisoning resistance of prepared catalysts, H_2_S and 1,2‐dichloroethane (typical emissions from petroleum refining and chemical industries) were introduced during MEK activity tests, respectively. In Figure 5a, under 10 ppm H_2_S exposure, the Pd/Co_3_O_4_ catalyst gradually deactivates until MEK conversion drops to 0%. Even after H_2_S removal, its activity fails to recover, indicating irreversible sulfur poisoning. In contrast, the Pd/CoN‐Co_4_N catalyst maintains ∼90% MEK conversion with negligible deactivation upon H_2_S exposure, and its performance remains stable after H_2_S is removed, demonstrating exceptional sulfur tolerance. Furthermore, when 100 ppm 1,2‐dichloroethane was introduced, Pd/CoN‐Co_4_N shows only a 5% decrease in MEK conversion, significantly outperforming the 14% decline observed for Pd/Co_3_O_4_ (Figure S27). After the inlet 1,2‐dichloroethane concentration increases to 150 ppm, the MEK conversions for Pd/CoN‐Co_4_N and Pd/Co_3_O_4_ decrease by 10% and 25%, respectively. Therefore, regardless of sulfur‐ or chlorine‐containing gas exposure, Pd/CoN‐Co_4_N exhibits superior poisoning resistance.

Beyond the electronic metal‐support interaction, the surface acid properties, particularly the abundance and strength of Lewis acid sites, are also crucial for the catalyst's poisoning resistance. We hypothesize that the strong EMSI plays a dual role in modulating the electronic structure of Pd and in tailoring the surface acid site distribution. To verify this, NH_3_ temperature‐programmed desorption (NH_3_‐TPD) was further employed to analyze the surface acidity and acid types of the catalysts. As shown in Figure 5b, NH_3_ desorption temperature regions can be classified into three categories: weak acid sites (< 200 °C), medium‐strong acid sites (200 °C–400 °C), and strong acid sites (> 400 °C), corresponding to terminal ─OH groups, Lewis acid sites, and Brønsted acid sites, respectively [60, 61, 62]. Acidic sites in all samples are primarily concentrated in the medium‐strong acid region. Notably, Pd/CoN‐Co_4_N shows the largest desorption peak area in this region, indicating the highest abundance of Lewis acid sites. Nitrogen incorporation alters the charge density distribution of Co species through electronegativity differences, effectively modulating both the strength and quantity of surface acid sites. Importantly, Lewis acid sites can polarize C─H or C─C bonds to reduce reaction activation energy, which exhibit enhanced electron‐pair accepting ability. The sulfur atom in H_2_S, possessing lone pair electrons and a highly polar S─H bond, acts as an electron donor. Similarly, the chlorine atoms in 1,2‐dichloroethane also contain lone pair electrons. The surface‐enriched strong Lewis acid sites on Pd/CoN‐Co_4_N act as sacrificial adsorption sites. Due to their electron‐deficient property, these Lewis acid sites exhibit a stronger affinity for H_2_S and 1,2‐dichloroethane molecules containing lone pair electrons, kinetically favoring their preferential adsorption. This effectively blocks their access to the Pd^0^ active centers. This electron competition adsorption mechanism enables Pd active sites to retain catalytic functionality under poisoning conditions.

H_2_S temperature‐programmed desorption with mass spectrometry (H_2_S‐TPD‐MS) and H_2_S‐MEK co‐adsorption models were constructed to investigate the poisoning resistance of the catalysts, using H_2_S as a representative poison. As shown in Figure S28, H_2_S‐TPD‐MS analysis reveals two H_2_S desorption peaks on both Pd/Co_3_O_4_ and Pd/CoN‐Co_4_N catalysts: a low‐temperature physical adsorption peak and a high‐temperature chemisorption peak. Pd/CoN‐Co_4_N exhibits lower desorption temperatures for both the physical (69 °C) and chemisorption (323 °C) peaks, indicating faster H_2_S desorption from the surface and reduced risk of persistent sulfur poisoning. However, if H_2_S initially adsorbs on noble metal centers, it can still directly poison active sites and deactivate the catalyst. To identify the adsorption locations of MEK and H_2_S, adsorption energies were calculated for co‐adsorption on Pd/Co_3_O_4_ and Pd/CoN‐Co_4_N models. Assuming MEK adsorbs on Pd active sites for activation, two configurations are simulated: both H_2_S and MEK co‐adsorbed on Pd sites, and H_2_S and MEK adsorbed separately on the support and Pd sites (Figure 5c). For Pd/Co_3_O_4_, the adsorption energy for co‐adsorption on Pd sites is 1.96 eV, while separate adsorption is −0.91 eV, suggesting a preference for H_2_S and MEK to compete for Pd active sites. In contrast, Pd/CoN‐Co_4_N shows lower adsorption energy for separate adsorption on the support and Pd sites (−2.58 eV) compared to co‐adsorption on Pd sites (−1.72 eV), indicating a spatially segregated adsorption mode where H_2_S binds to the support and MEK to Pd sites. This adsorption selectivity originates from the redistribution of Co species’ electron density induced by the EMSI. The enriched Lewis acid sites on the cobalt nitride support preferentially capture H_2_S lone pair electrons *via* enhanced electron‐accepting properties of Co sites, to form Co─S coordination bonds. The synergistic effects of spatial hindrance and electronic competition adsorption mechanisms enable Pd active centers to maintain efficient MEK activation ability even under sulfur poisoning conditions.

To investigate the intrinsic effects of H_2_S and 1,2‐dichloroethane on catalytic performance during MEK oxidation, *in situ* DRIFTS tests were conducted toward Pd/Co_3_O_4_ and Pd/CoN‐Co_4_N catalysts with the introduction of H_2_S and 1,2‐dichloroethane to observe changes in peaks of intermediates. As shown in Figure 5d, introducing H_2_S during MEK reaction on Pd/Co_3_O_4_ significantly enhances intermediate product peaks, particularly carboxylate and carbonate species, while the C═O vibration peak of MEK at 1744 cm^−1^ markedly decreases. In contrast, the vibration peaks of Pd/CoN‐Co_4_N remain nearly unaffected by H_2_S introduction. Figures S29 and S30 reveal that adding 100 ppm 1,2‐dichloroethane during MEK oxidation on Pd/Co_3_O_4_ notably intensifies intermediate product peaks and reduces MEK adsorption vibration peaks. After removing 1,2‐dichloroethane, the infrared peaks largely recover to their original levels. However, reintroducing a higher concentration of 1,2‐dichloroethane (150 ppm) further increases intermediate peaks and suppresses MEK adsorption. For Pd/CoN‐Co_4_N, MEK adsorption is only impacted at 150 ppm 1,2‐dichloroethane.

These results suggest that the inferior poisoning resistance of Pd/Co_3_O_4_ is due to competitive adsorption and diminished oxidation ability, leading to intermediate accumulation (Figure 5e). However, Pd/CoN‐Co_4_N benefits from abundant Lewis acid sites generated *via* strong EMSI. The lone pair electrons in H_2_S and 1,2‐dichloroethane preferentially adsorb on acid sites and protect Pd active sites from damage. Simultaneously, optimized interfacial charge transfer abilities accelerate deep oxidation of intermediates and further enable sustained high catalytic efficiency under a poisoning environment.

## 3 Conclusion

Oxygenated volatile organic compounds (OVOCs) are key contributors to atmospheric pollution, among which methyl ethyl ketone (MEK) is extensively used as an indispensable solvent across various industrial applications. Industrial exhaust gases often contain impurities such as H_2_S and 1,2‐dichloroethane, which can form surface coverage layers on the active sites of noble metal catalysts or induce competitive chemisorption, thereby obstructing reaction pathways. Beyond merely diminishing catalytic activity, these effects have the potential to lead to catalyst deactivation. Therefore, the development of highly efficient and stable catalysts for the low‐temperature oxidation of MEK remains a significant challenge.

In this work, catalysts with varying EMSI strengths were rationally constructed through a differential nitrogen modulation of the support. Among them, Pd/CoN‐Co_4_N with the strongest EMSI achieves 90% of MEK conversion at just 172 °C. The EMSI in Pd/CoN‐Co_4_N enhances interfacial charge transfer ability, enabling more electrons to migrate from Co to Pd sites at the interface without reductants, thereby in situ generating abundant Pd^0^. This effectively increases active sites, significantly enhances the adsorption and activation efficiency of MEK and O_2_ molecules, and accelerates the deep oxidation of carboxylate intermediates. Additionally, the catalytic performance of Pd/CoN‐Co_4_N remains nearly unaffected in the presence of trace H_2_S and 1,2‐dichloroethane. The strong EMSI promotes more Lewis acid sites generated by modifying the charge density of Co atoms in the support, which preferentially bond with molecules possessing lone pair electrons, thereby protecting active sites and effectively addressing the susceptibility of noble metal catalysts to poisoning. This study elucidates the regulatory mechanism of EMSI between noble metals and non‐oxide supports on the electronic structure of active centers, reveals the intrinsic mechanism for enhancing catalytic performance and poisoning resistance, and provides a theoretical foundation for designing heterogeneous catalysts toward complex industrial atmospheric conditions.

## Author Contributions

The manuscript was written through the contributions of all authors. C.H., Y.W., and C.A. conceived the experiments. C.W. supervised the project and conducted the data curation. Y.W. and C.A. performed the preparation of materials as well as carried out the experiments of performance evaluation and characterizations. Z.J. and J.W. performed the operando experiments. J.W. and X.F. contributed to the theoretical studies and assisted with analyzing the results. X.F. and C.H. helped to carry out the characterizations. All authors discussed these results and reviewed the manuscript.

## Funding

This work was funded by the National Natural Science Foundation of China (22276145, 22476157, 22406146, 22206153), China Postdoctoral Science Foundation (2023M732783), and Basic Scientific Research Fees of Central Universities, Natural Science Basic Research Plan in Shaanxi Province of China (2025JC‐YBQN‐497).

## Conflicts of Interest

The authors declare no competing financial interest.

## Supporting information




**Supporting File**: advs75899‐sup‐0001‐SuppMat.docx.

## Data Availability

The datasets generated or analyzed during the current study are available from the corresponding author upon reasonable request.
