# Supplementary material for: Metal‐Support Interaction Triggered Electronic Reconstruction in Pd/CoN‐Co4N Catalysts for Optimizing MEK Oxidation and Poisoning Resistance
Source: Adv Sci (Weinh). 2026 Jun 2:e75899. Online ahead of print. doi: 10.1002/advs.75899 (PMC13336818; doi:10.1002/advs.75899)
Supplement: Supplementary file 1 — Supporting File: advs75899‐sup‐0001‐SuppMat.docx [file ADVS-9999-e75899-s001.docx]

**Supporting Information**

Metal-Support Interaction Triggered Electronic Reconstruction in Pd/CoN-Co_4_N Catalysts for Optimizing MEK Oxidation and Poisoning Resistance

*Yadi Wang^1^, Chaoqian Ai^1^, Cai Wang^1^, Zeyu Jiang, Jialei Wan, Jingjing Wang, Xiangbo Feng^*^, Chi He^*^*

**Summary: 46 pages, 30 Figures, and 3 Tables.**

**Table of Contents**

**Catalyst Preparation S3**

**Catalyst Characterizations S3**

**Catalytic Performance Evaluation S6**

***In situ* DRIFTS S8**

**Theoretical Investigations S9**

**Supplementary Figures S12**

**Supplementary Tables S42**

**References S45**

**Catalyst Preparation**

**Synthesis of** **Co_3_O_4_**

Typically, Co_3_O_4_ support was directly synthesized *via* the pyrolysis method by calcining cobalt acetate in air, with a heating rate of 2 °C·min^−1^ from RT to 500 °C for 2 h.

**Synthesis of Co-N**

Different structured cobalt nitrides were synthesized *via* a nitridation method. The Co_3_O_4_ support was further nitridated under an NH_3_ atmosphere at temperatures of 300, 500, and 700 °C with a heating rate of 5 °C·min^−1^ for 3 h, yielding Co-N supports with distinct structures (CoN and Co_4_N).

**Synthesis of Pd/Co_3_O_4_, Pd/Co_3_O_4_-CoN, Pd/CoN-Co_4_N and Pd/Co_4_N**

In brief, the loading of noble metal palladium was performed using the rotary evaporation method. Specifically, 0.5 g of the support was dissolved in 40 mL of ultrapure water and ultrasonicated for 30 min to achieve uniform dispersion. Simultaneously, Na_2_PdCl_4_ was dissolved in 10 mL of ultrapure water to form a homogeneous yellow solution, which was then directly poured into the support solution. The mixture was subjected to rotary evaporation at 60 °C until complete dryness. The resulting powder was washed with deionized water and ethanol, dried overnight at 80 °C, and finally calcined at 350 °C under N_2_ flow for 3 h. The obtained products were labeled as Pd/Co_3_O_4_, Pd/Co_3_O_4_-CoN (the support nitrided at 300 °C), Pd/CoN-Co_4_N (the support nitrided at 500 °C), and Pd/Co_4_N (the support nitrided at 700 °C).

**Catalyst Characterizations**

The high-resolution transmission electron microscope (HR-TEM) images were recorded on the G2-F30 (FEI, America) microscope operating at an acceleration voltage of 300 kV. An oxfordmax-80 energy-dispersive X-ray detector, mounted in the above HR-TEM, was used to record EDS-mapping images. A JSM-7401F (Japan) was used to obtain the scanning electron microscope (SEM) images. X-ray diffraction (XRD) patterns were acquired using a powder X-ray diffractometer (PANalytical X’pert MPD Pro, Netherlands) with Ni-filtered Cu-*K*_α_ irradiation. The tube voltage was 40 kV, and the current was 40 mA. The XRD patterns were obtained in the 2θ range of 10–80° with a scanning rate of 10°·min^−1^. The actual Pd content of samples was determined by an ICPE-9000 (SHIMADZU, Japan) apparatus. A Thermo Scientific *K*-Alpha (America) with a monochromatized Al-*K*_α_ line source was used to carry out the X-ray photoelectron spectroscopy (XPS) measurement. Binding energies were calibrated using the containment carbon (C 1*s* = 284.8 eV). Bruker Vetex70 (Germany) and LabRAM HR (Horiba Jobin Yvon) were used to obtain the samples’ molecular structure. Raman spectra were conducted at *λ* = 532 nm. The FTIR spectra were obtained using the KBr pressing method with an FT-IR spectrometer (Nicolet iS10, Thermo Fisher Scientific, America) at a resolution of 4 cm^−1^ in the range of 4000-400 cm^−1^.

*In situ* DRIFTS of CO adsorption (CO-DRIFTS) were conducted on a Bruker Tensor 37 equipped with an MCT detector in the range of 4000-600 cm^−1^ with a resolution of 4 cm^−1^ and 64 acquisition scans. The catalyst powder was put in an *in situ* IR cell equipped with a KBr window. Before adsorption, *in situ* passivation (200 °C for 60 min in N_2_ condition; 100 mL·min^−1^) pretreatment. The cell was then cooled down to 25 °C in an N_2_ flow (100 mL·min^−1^). The catalyst itself was used as the background of the spectra. Following this, 10% CO/N_2_ (50 mL·min^−1^) gas was introduced into the cell and adsorbed for at least 60 min, where it was observed that the bands associated with CO adsorption on Pd species no longer changed. The system was flushed for 20 min in the N_2_ stream (100 mL·min^−1^) to remove any gas-phase CO.

Temperature-programmed reduction of hydrogen (H_2_-TPR) experiments was performed on a PCA-1200 (Builder, China) equipped with a TCD detector. 100 mg of catalyst was fixed in the U-type quartz tube and pretreated at 200 °C (10 °C·min^−1^) for 60 min under N_2_ with a flow rate of 30 mL·min^−1^. Then the samples were reduced from RT to 900 °C with a heating rate of 10 °C·min^−1^ under a mixture of 5% H_2_/Ar (30 mL·min^−1^). Hydrogen consumption was measured online using a thermal conductivity detector.

Temperature-programmed desorption of oxygen (O_2_-TPD) experiment was performed on a Builder PCA-1200 analyzer equipped with a TCD detector. Typically, 150 mg of catalyst was fixed in the U-type quartz tube and pretreated in a pure N_2_ flow (40 mL·min^−1^) at 200 °C for 60 min, which was cooled down to room temperature followed by the adsorption of O_2_ for 90 min. After being saturated with O_2_, the catalyst was flushed with a pure N_2_ flow (40 mL·min^−1^) for 30 min at room temperature. The desorption profiles of O_2_-TPD were recorded online with a heating rate of 10 °C·min^−1^.

Temperature-programmed desorption of MEK (MEK-TPD) experiment was performed on a Builder PCA-1200 analyzer equipped with a TCD detector. Typically, 150 mg of catalyst was fixed in the U-type quartz tube and pretreated in a pure N_2_ flow (40 mL·min^−1^) at 200 °C for 60 min, which was cooled down to room temperature followed by the adsorption of MEK/N_2_ for 90 min. After being saturated with MEK, the catalyst was flushed with a pure N_2_ flow (40 mL·min^−1^) for 30 min at room temperature. The desorption profiles of MEK-TPD were recorded online with a heating rate of 10 °C·min^−1^.

Temperature-programmed desorption of ammonia (NH_3_-TPD) experiment was performed on a Builder PCA-1200 analyzer equipped with a TCD detector. Typically, 150 mg of catalyst was fixed in the U-type quartz tube and pretreated in a pure N_2_ flow (40 mL·min^−1^) at 200 °C for 60 min, which was cooled down to 50 °C followed by the adsorption of 5% NH_3_/N_2_ for 90 min. After being saturated with NH_3_, the catalyst was flushed with a pure N_2_ flow (40 mL·min^−1^) for 30 min at 50 °C. The desorption profiles of NH_3_-TPD were recorded online with a heating rate of 10 °C·min^−1^.

H_2_S temperature-programmed desorption with mass spectrometry was performed using a fixed bed microreactor connected to a mass analyzer (H_2_S-TPD-MS). Initially, 50 mg of the catalyst was taken and purged with He at room temperature until the signal was stabilized. Then started at a rate of 10 °C·min^−1^ from RT to 600 °C under an Ar flow. The effluent gases were analyzed using an online mass spectrometer (HENGPING, China), and the signals at mass-to-charge (m/z) ratios of 34 (H_2_S) were detected.

Temperature-programmed surface reaction (TPSR) of MEK was performed on the chemisorption analyzer (PCA-1200-MS) connected to a mass analyzer. To adapt the instrument cannot be used below room temperature, the catalyst usage here was reduced for tests. About 50 mg catalyst was fixed in the U-type quartz tube and pre-heated to 200 °C at a heating rate of 10 °C·min^−1^ in a He atmosphere of 30 °C·min^−1^ for 60 min, and then naturally cooled to room temperature. The mixed gas same as the reaction flow was introduced into the tube. Followed by keeping at this condition for 60 min to reach the balance of adsorption and desorption, and then the tube was heated from room temperature to 300 °C with a heating rate of 2 °C·min^−1^. The products in the exhaust gas were recorded by a SHP8400PMS-L mass spectrometer (HENGPING, China).

**Catalytic Performance Evaluation**

The prepared catalysts were tested for MEK oxidation. The performance evaluation processes were conducted in a fixed bed catalytic reactor with accurately controlled temperature and gas flow. The materials were tableted and sieved to 40-60 mesh before evaluation. In each test, 0.3 g of sample was fixed in the tube reactor (6 mm) by silica wool. The ready-state MEK oxidation activity was measured, with the duration time for each temperature point maintained as 30 min. The MEK feed (1000 ppm) was generated using N_2_ bubbler in an ice bath at 15 °C and mixed with simulated air (79% N_2_ + 21% O_2_). The total flow rate was controlled at 150 mL·min^−1^ thus achieving the WHSV of 30,000 mL·g^−1^·h^−1^. Considering the effect of H_2_S and 1,2-dichloroethane on the catalytic activity, MEK oxidation experiments were also performed in the presence of 10 ppm H_2_S, 100 ppm and 150 ppm 1,2-dichloroethane.

The catalyst bed was subsequently set to the desired temperature and left to equilibrate for 30 min before online sampling was initiated. The concentrations of MEK and CO_2_ were monitored by an on-line gas chromatograph (GC-9890A, Shanghai Sida, China) equipped with a flame ionization detector (FID) and a methane converter in front of a flame ionization detector (FID). The methane converter is a micro high-temperature furnace (350 °C) with Ni-based catalyst that can fully convert CO and CO_2_ into CH_4_ for FID detection. The specific catalytic performance evaluation methods were given.

The conversion of MEK (*X*_MEK_), CO_2_ yield (*Y*_CO2_), and selectivity of CO_2_ (*Z*_CO2_) were calculated as Eqs (S1-S3),

$X_{\text{MEK}}\text{(\%)=}\frac{\left[\text{MEK}\right]\text{in-}\left[\text{MEK}\right]\text{out}}{\left[\text{MEK}\right]\text{in}}\times\text{100\%}$ (S1)

$Y_{\text{CO}_{2}}(\%)=\frac{\left[\text{CO}_{2}\right]\text{out}}{\left[\text{MKE}\right]\text{in}}\times100\%$ (S2)

$Z_{\text{CO}_{2}}\text{(\%)=}\frac{\left[\text{CO}_{2}\right]_{\text{out}}}{\left[\text{MEK}\right]_{\text{in}}-\left[\text{MEK}\right]_{\text{out}}}\times\text{100\%}$ (S3)

where [MEK]_in_ and [MEK]_out_ represent the MEK concentrations in the inlet and outlet gases, respectively; [CO_2_]_out_ is the concentration of outlet CO_2_.

The reaction rate (*r*_MEK_, mol·g_Pd_^−1^·s^−1^) was calculated as Eq (S4),

$r_{\text{MEK}}=\frac{X_{\text{MEK}}\text{·}V_{\text{MEK}}}{W_{\text{cat}}\text{·}w\%_{\text{Pd}}}$ (S4)

where *W*_cat_ represents the catalyst weight (g), *w*%_Pd_ is the content of Pd in catalyst (%), and *V*_MEK_ is the MEK gas flow rate (mol·s^−1^).

The empirical kinetic expression of the reaction rate equation for MEK oxidation can be described as Eq (S5),

$r_{\text{MEK}}=A\exp\left(-\frac{E_{\text{a}}}{RT}\right)P_{\text{MEK}}^{\alpha}P_{\text{O}_{2}}^{\beta}$ (S5)

Taking the natural logarithm of Eq (S5), Eq (S6) can be obtained,

$\ln r=\ln A+\alpha\ln P_{\text{MEK}}+\beta\ln P_{\text{O}_{2}}-E_{\text{a}}/\left(RT\right)$ (S6)

The components of the reactant gas feed undergo minor changes during the kinetics data testing. Therefore, ln *A*, *α*ln *P*_MEK_, *β*ln *P*_O2_ can be supposed to be approximately constant, and Eq (S6) can be simplified to Eq (S7),

$\ln r=-\frac{E_{\text{a}}}{RT}+C$ (S7)

The apparent activation energy (*E*_a_) can be obtained from the slope of the resulting linear plot of ln*r* versus 1/*T*.

***In situ* DRIFTS**

*In situ* DRIFTS for MEK oxidation over synthesized samples were collected from the Tensor 37 (Bruker, Germany) infrared spectrometer containing an MCT detector. The liquid nitrogen was adopted to cool this detector. The *in situ* reaction cell covered by KBr window was connected to the gas flow control system. Around 30 mg of the sample was placed into the cell and then pretreated to purify its surface through a calcination process in N_2_ at 200 °C for 60 min with a gas flow of 100 mL·min^−1^. During the cooling process, the backgrounds were determined over the original catalyst surface under N_2_ flow at each selected temperature point. Following, the mixed gas of 1000 ppm MEK containing 21 vol.% O_2_/N_2_ was introduced into the reaction cell with a gas flow of 150 mL·min^–1^ in the range of 100-220 °C. After maintaining for 30 min at each temperature point to achieve stabilization, the spectra were recorded at a resolution of 4 cm^−1^ in the range of 4000-600 cm^−1^ with 100 scans. In addition, the *in situ* DRIFTS were further recorded under different atmospheres (N_2_/Air) and different volumes of water vapor for each catalyst at the selected temperature.

**Theoretical Investigations**

We have employed the Vienna Ab Initio Package (VASP) to perform all the density functional theory (DFT) calculations within the generalized gradient approximation (GGA) using the PBE formulation.^1-3^ We have chosen the projected augmented wave (PAW) potentials to describe the ionic cores and take valence electrons into account using a plane wave basis set with a kinetic energy cutoff of 500 eV.^4,5^ Partial occupancies of the Kohn−Sham orbitals were allowed using the Gaussian smearing method and a width of 0.03 eV. The electronic energy was considered self-consistent when the energy change was smaller than 10^−5^ eV. A geometry optimization was considered convergent when the force change was smaller than 0.05 eV/Å. Grimme’s DFT-D_3_ methodology was used to describe the dispersion interactions.^6^ During structural optimizations, the Γ point in the Brillouin zone was used for k-point sampling, and all atoms were allowed to relax. Finally, the adsorption energies (*E*_ads_) were calculated as *E*_ads_= *E*_ad_/_sub_ -*E*_ad_ -*E*_sub_, where *E*_ad/sub_, *E*_ad_, and *E*_sub_ are the total energies of the optimized adsorbate/substrate system, the adsorbate in the structure, and the clean substrate, respectively. The free energy of a gas phase molecule or an adsorbate on the surface was calculated by the equation G = E + ZPE − TS, where E is the total energy, ZPE is the zero-point energy, T is the temperature in kelvin (298.15 K is set here), and S is the entropy. Finally, we define the formation energy per atom (*E*_f_) of a compound, where the dopant $E_{\text{f}}=E_{\text{total}}-N_{\text{Co}}\mu_{\text{Co}}-N_{\text{Pd}}\mu_{\text{Pd}}-N_{\text{O}}\mu_{\text{O}}-N_{\text{N}}\mu_{\text{N}}$, where *E*_total_ is the total DFT energy of a given structure, and *μ*_{Co, Pd, O, N}_ are the chemical potentials of the constituent atomic species, *N*_{Co, Pd, O, N}_ are the number of the constituent atomic species.

The equilibrium lattice constants of CoN, Co_4_N, Co_3_O_4_ unit cell were optimized. We then use it to construct a CoN, Co_4_N, Co_3_O_4_(111) spercell surface model. During structural optimizations, a 3 × 3 × 1 *k*-point grid in the Brillouin zone was used for *k*-point sampling, and the bottom two atomic layers were fixed while the others were allowed to relax. The CoN-Co_4_N interface structures had been established using the CoN and Co_4_N surface with the lattice parameters (a = 11.2142 Å, b = 14.4977 Å, c = 25.6652 Å), and the mismatch rate is 4.89%. The Pd/Co_3_O_4_ structures with 5-layer of Co-O layers include the 13 Pd atoms with the lattice parameters (a = 11.3786 Å, b = 11.3786 Å, c = 25.7459 Å). Pd/CoN-Co_4_N had been established using the CoN-Co_4_N interface and 13 Pd atoms with the lattice parameters the lattice parameters (a = 11.2142 Å, b = 14.4977 Å, c = 25.6652 Å).

The DFT calculations in this work are intended to reveal the qualitative trends of electronic interactions between different supports and Pd. The models employed are idealized periodic surfaces and do not account for more complex factors potentially present in real catalysts, such as adsorption at high coverages, surface defects, or dynamic reconstruction under reaction temperatures.

**
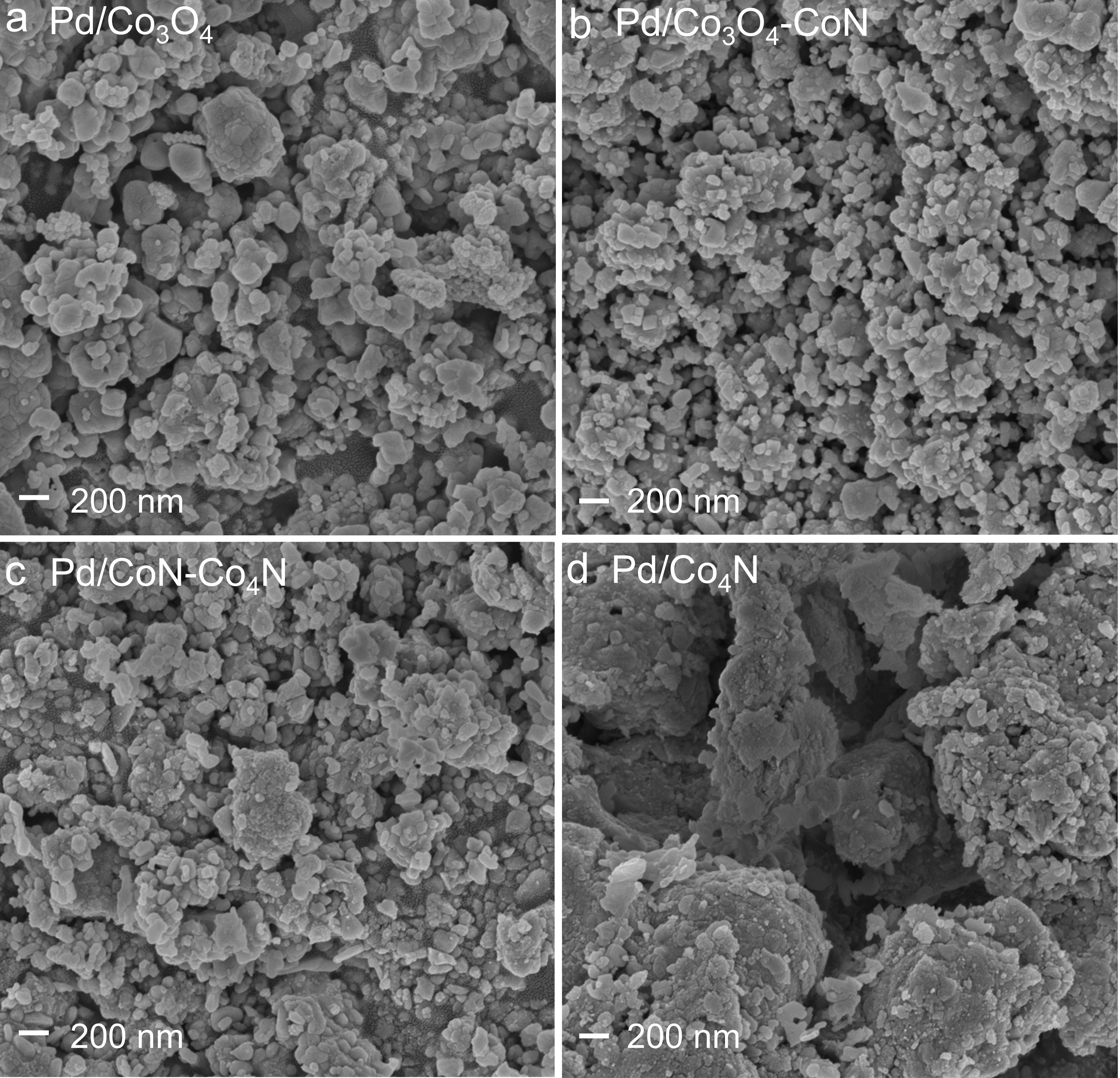
**

**Figure S1**. SEM images of (a) Pd/Co_3_O_4_, (b) Pd/Co_3_O_4_-CoN, (c) Pd/CoN-Co_4_N, and (d) Pd/Co_4_N catalysts.

**Figure S2.** HR-TEM images of (a) Pd/Co_3_O_4_, (b) Pd/Co_3_O_4_-CoN, (c) Pd/CoN-Co_4_N, and (d) Pd/Co_4_N catalysts.


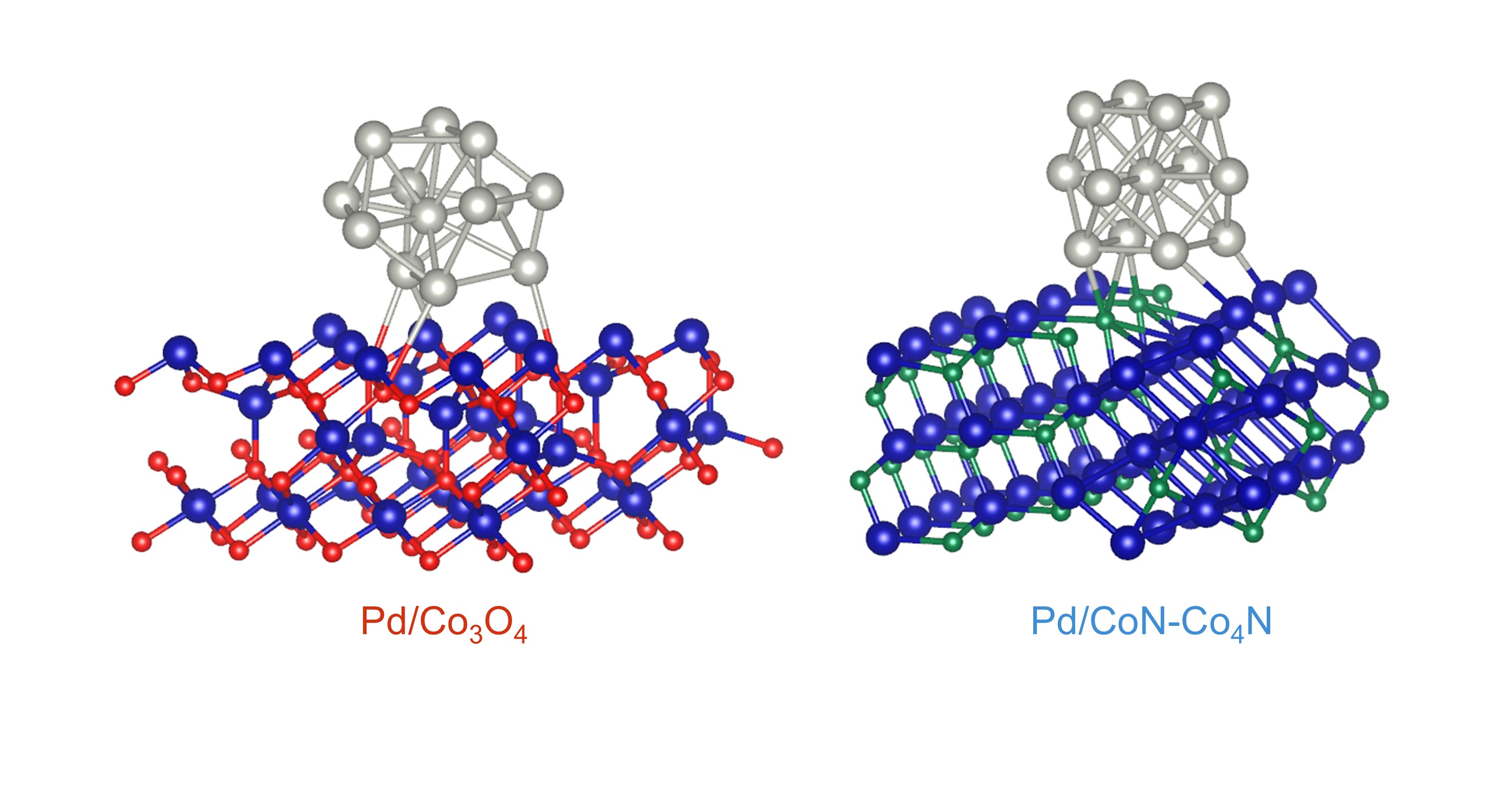


**Figure S3.** The optimistic structures of Pd/Co_3_O_4_ and Pd/CoN-Co_4_N catalysts defined by DFT calculations (color code: Co (blue), O (red), Pd (gray), and N (green)).


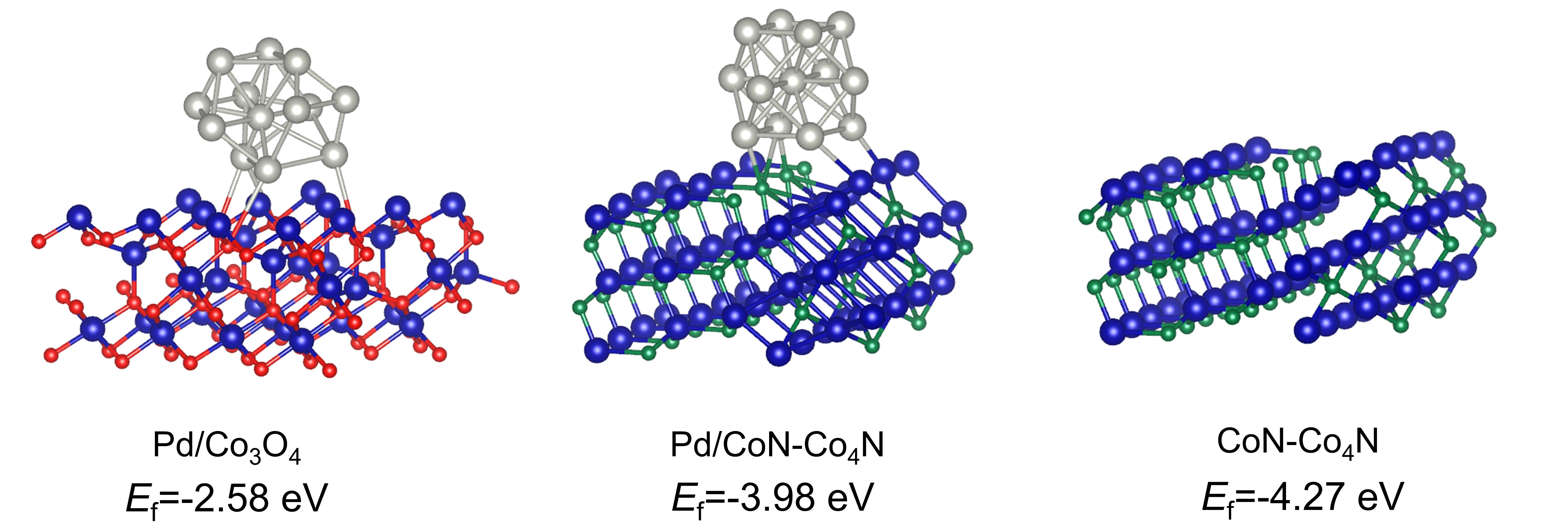


**Figure S4.** The formation energy of Pd/Co_3_O_4_, Pd/CoN-Co_4_N catalysts and CoN-Co_4_N heterojunction (color code: Co (blue), O (red), Pd (gray), and N (green)).

**
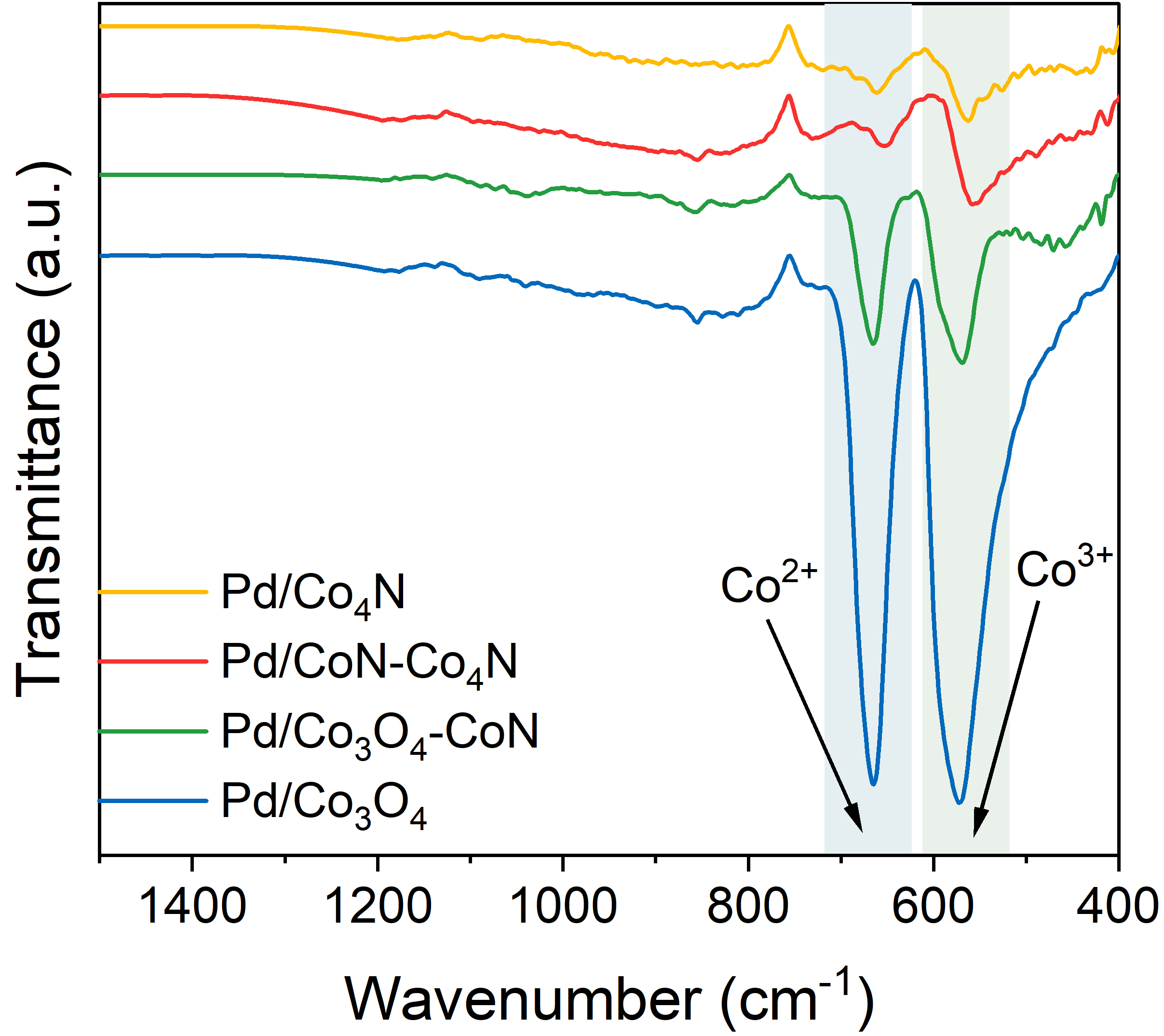
**

**Figure S5.** FT-IR spectra of supported-Pd catalysts.

**
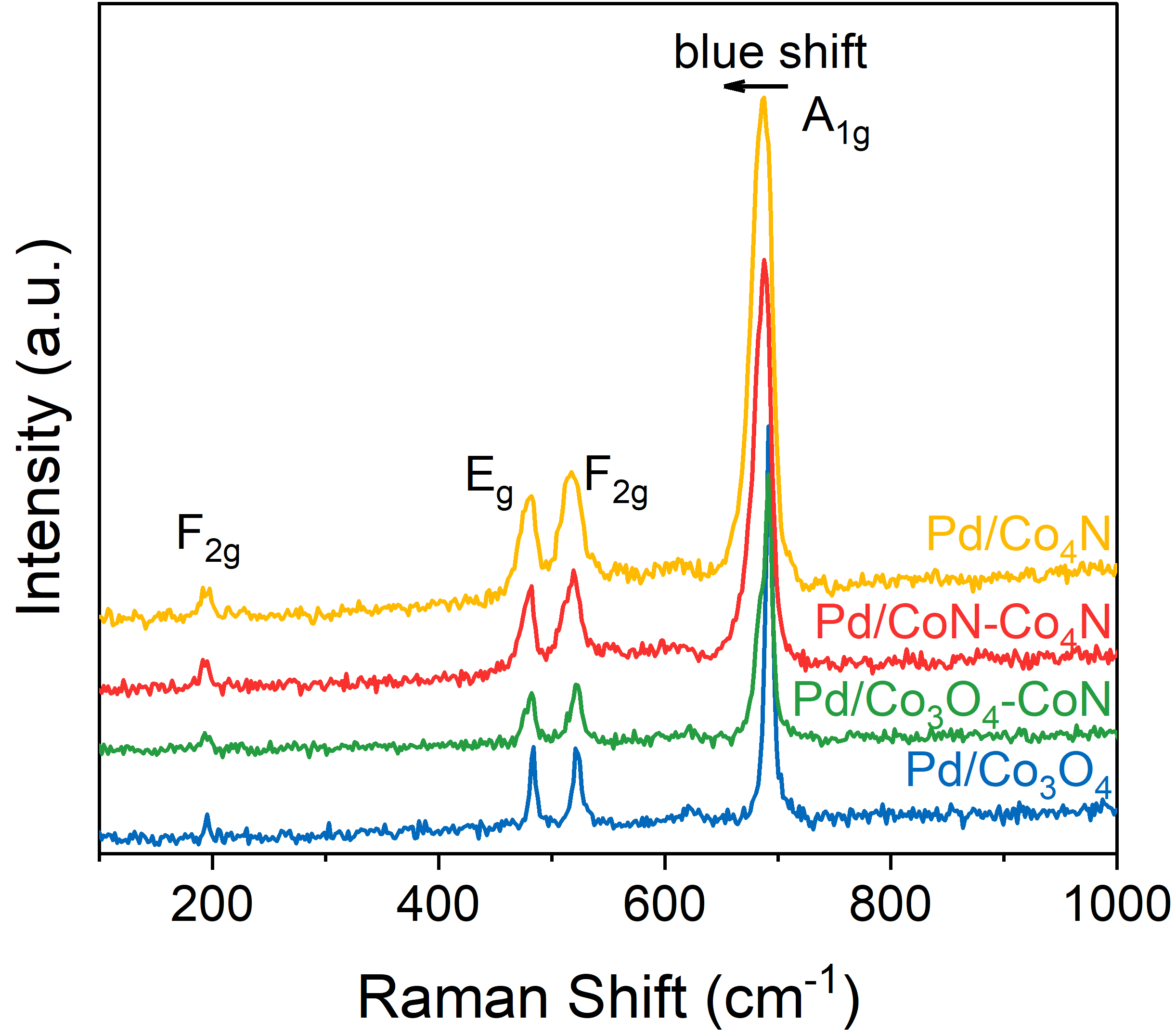
**

**Figure S6.** Raman spectra of supported-Pd catalysts.

**
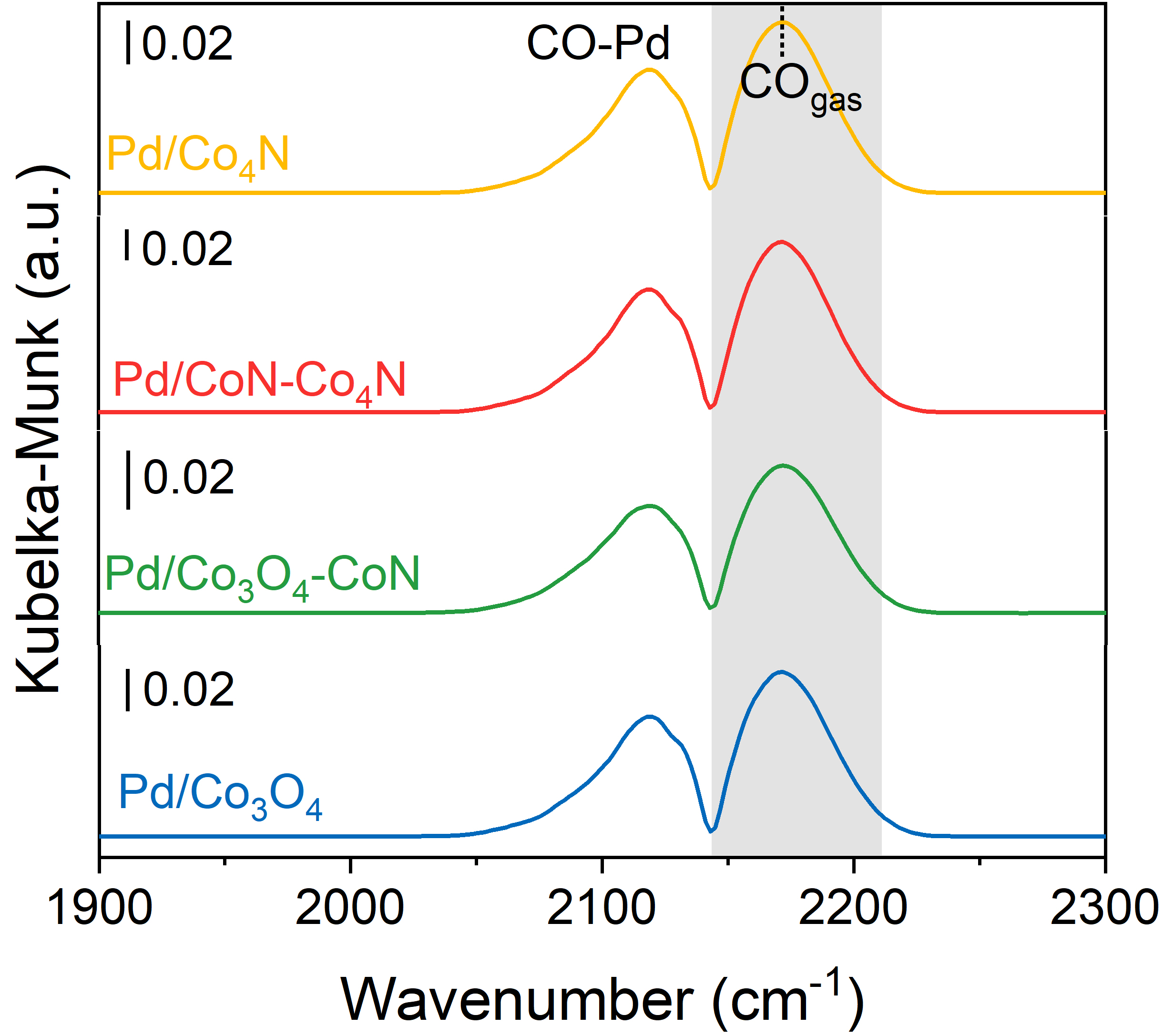
**

**Figure S7.** CO-DRIFTS of supported-Pd catalysts.

**
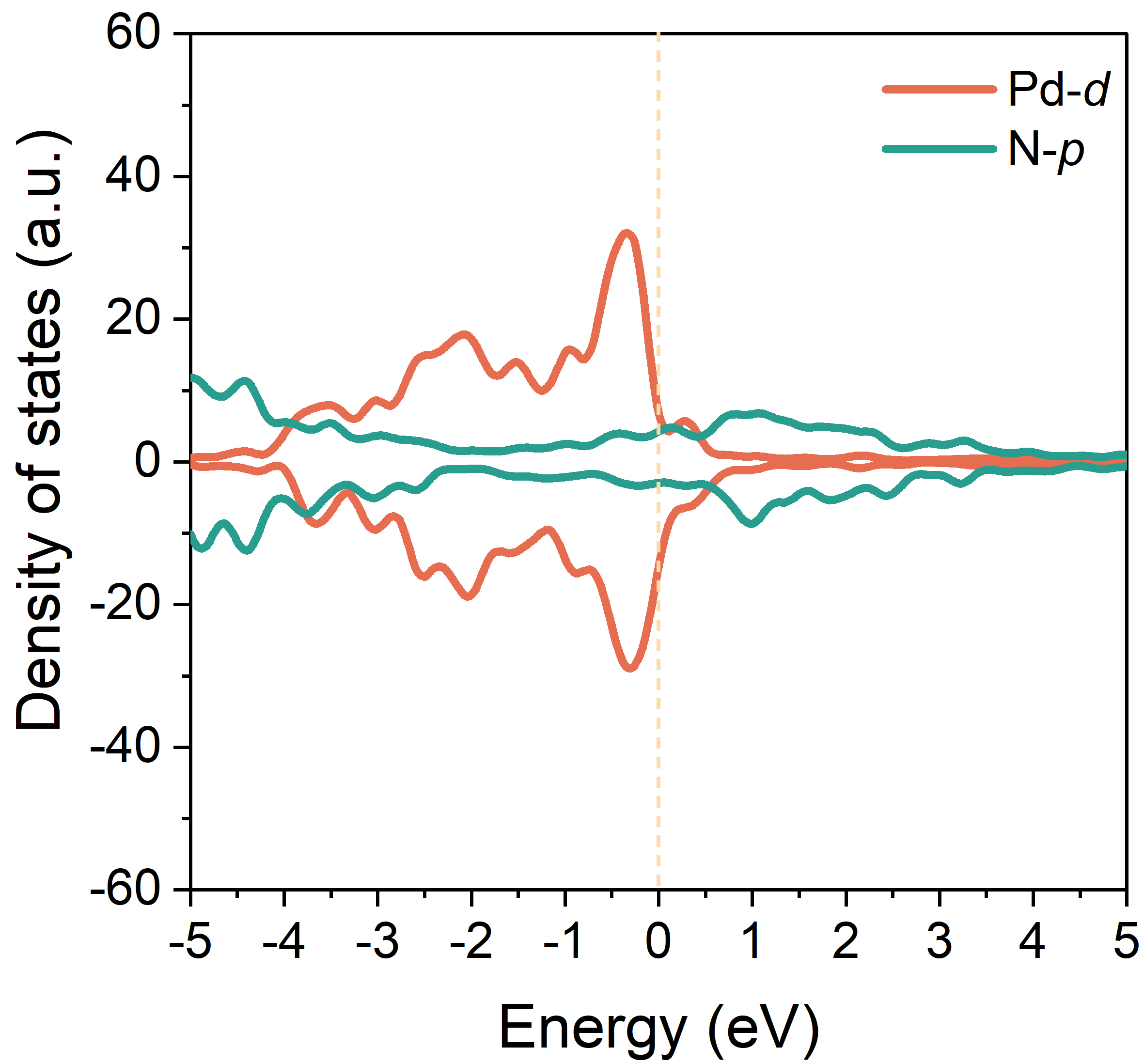
**

**Figure S8.** The PDOS of Pd and N over Pd/CoN-Co_4_N.

**
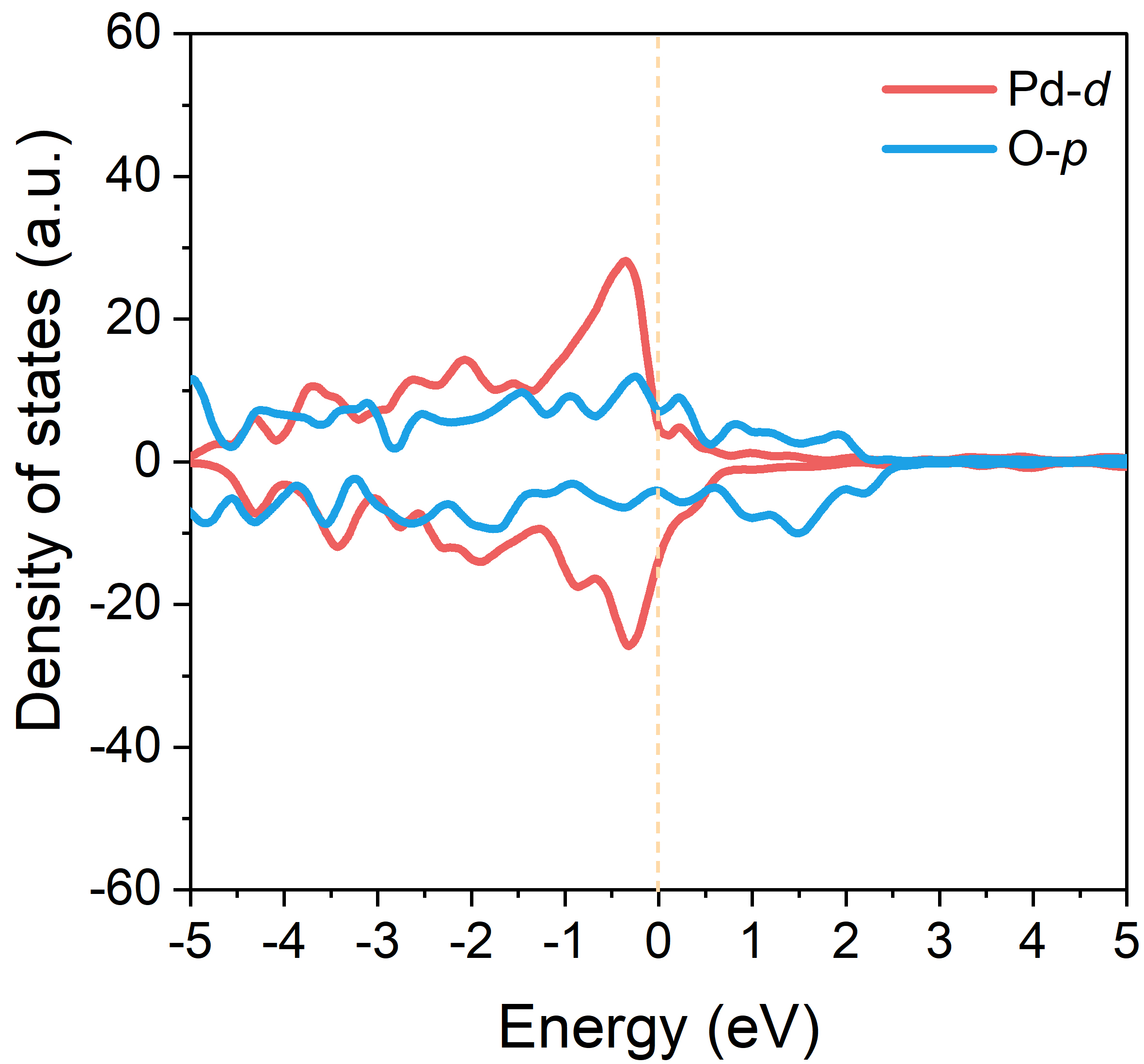
**

**Figure S9.** The PDOS of Pd and O over Pd/Co_3_O_4_.

**
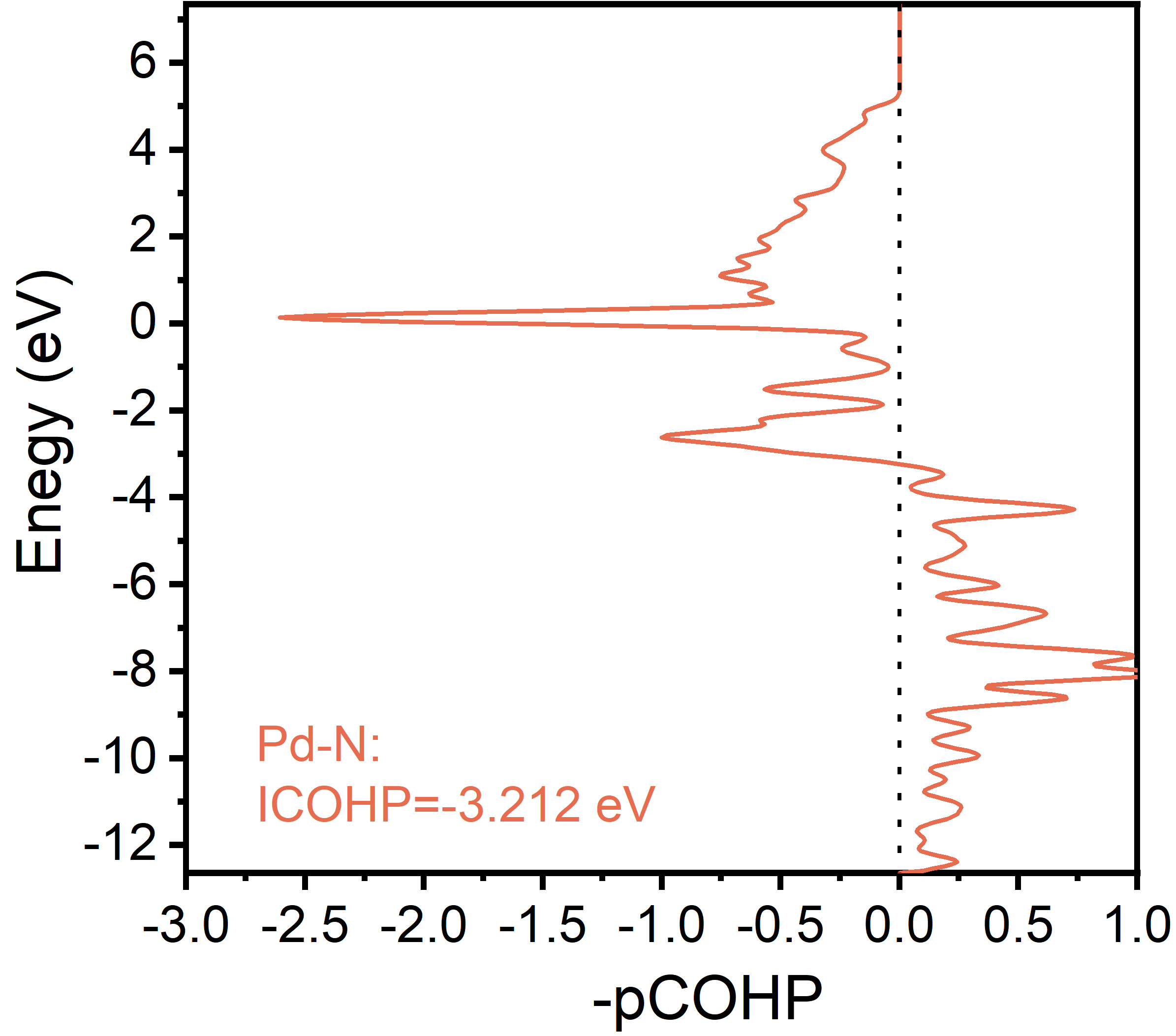
**

**Figure S10.** COHP analysis of the Pd-N bond over Pd/CoN-Co_4_N.

**
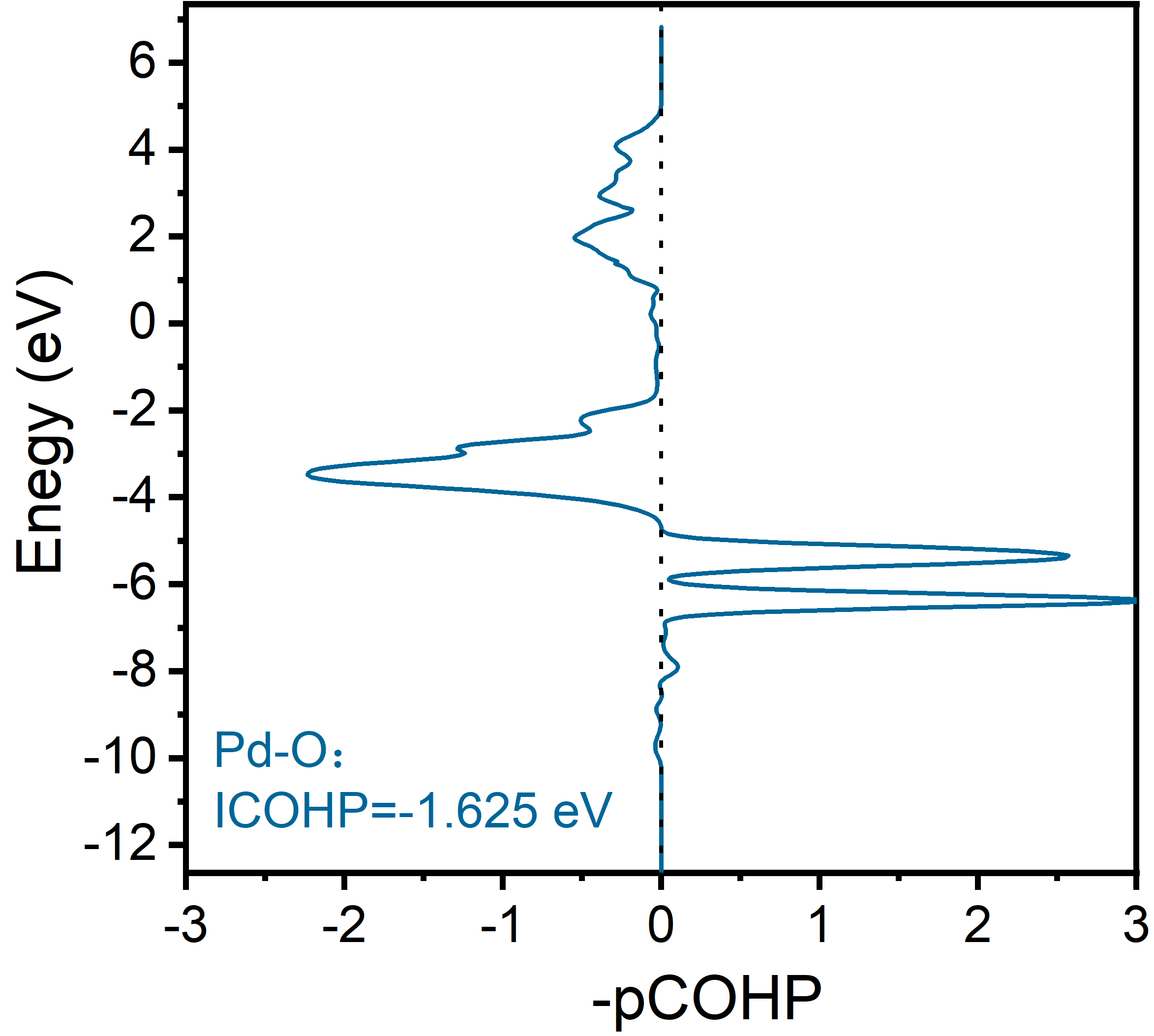
**

**Figure S11.** COHP analysis of the Pd-O bond over Pd/Co_3_O_4_.

**
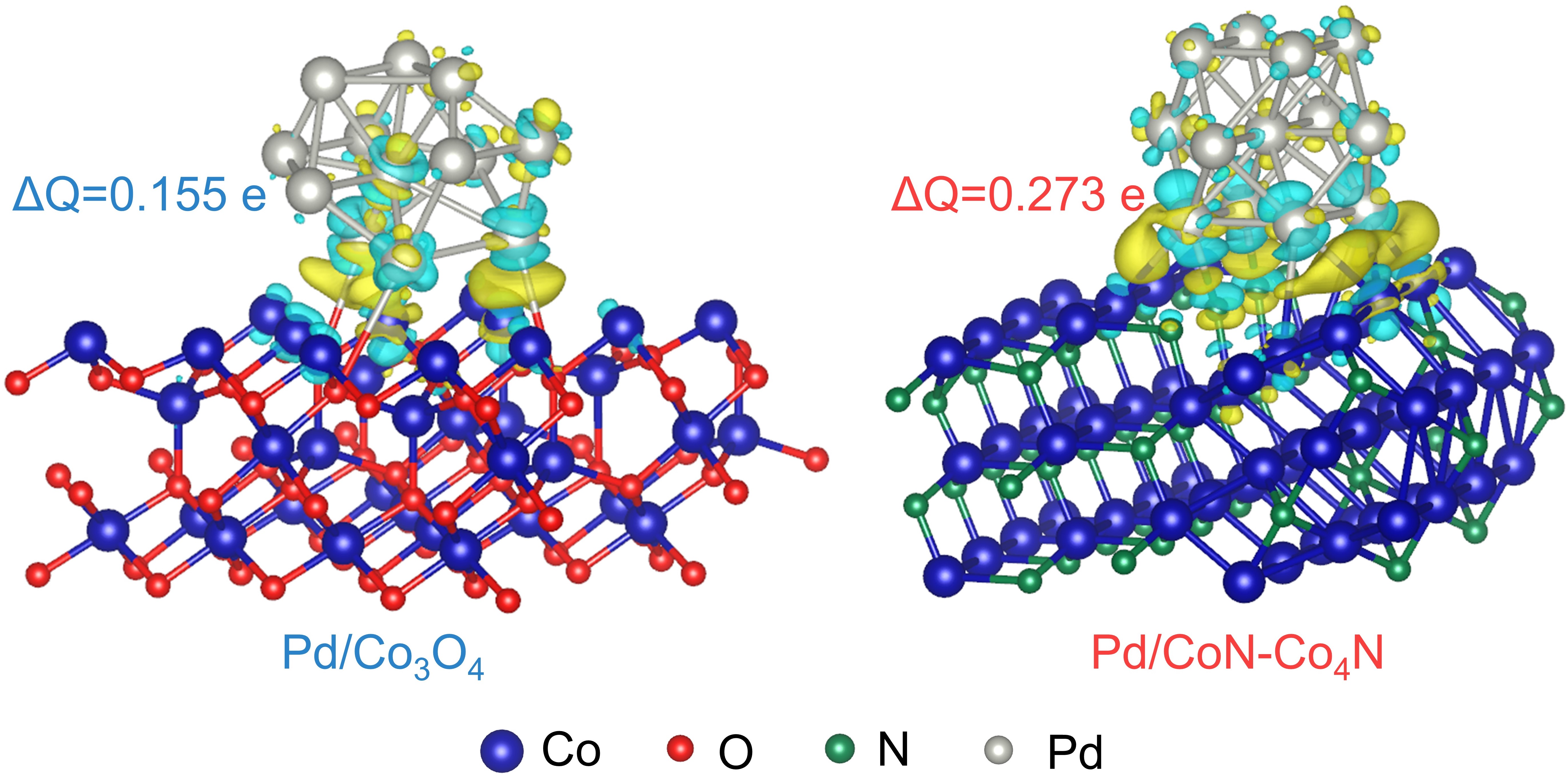
**

**Figure S12.** Calculated charge density differences of Pd/Co_3_O_4_ and Pd/CoN-Co_4_N catalysts (color code: Co (blue), O (red), Pd (gray), and N (green); the yellow and cyan iso-surfaces represent charge accumulation and depletion in space, respectively).

**
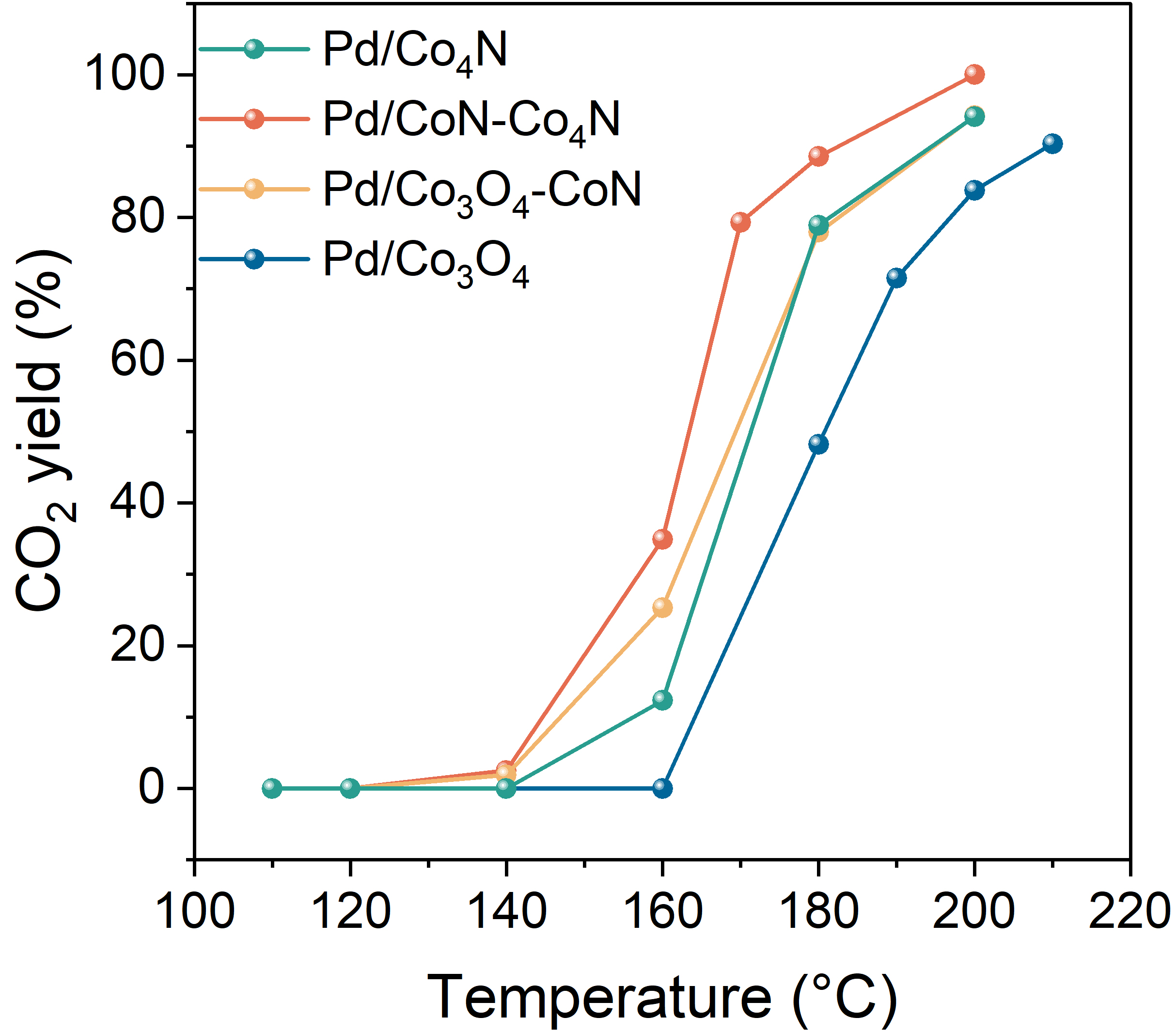
**

**Figure S13.** CO_2_ yield of supported-Pd catalysts for the oxidation of MEK.

**
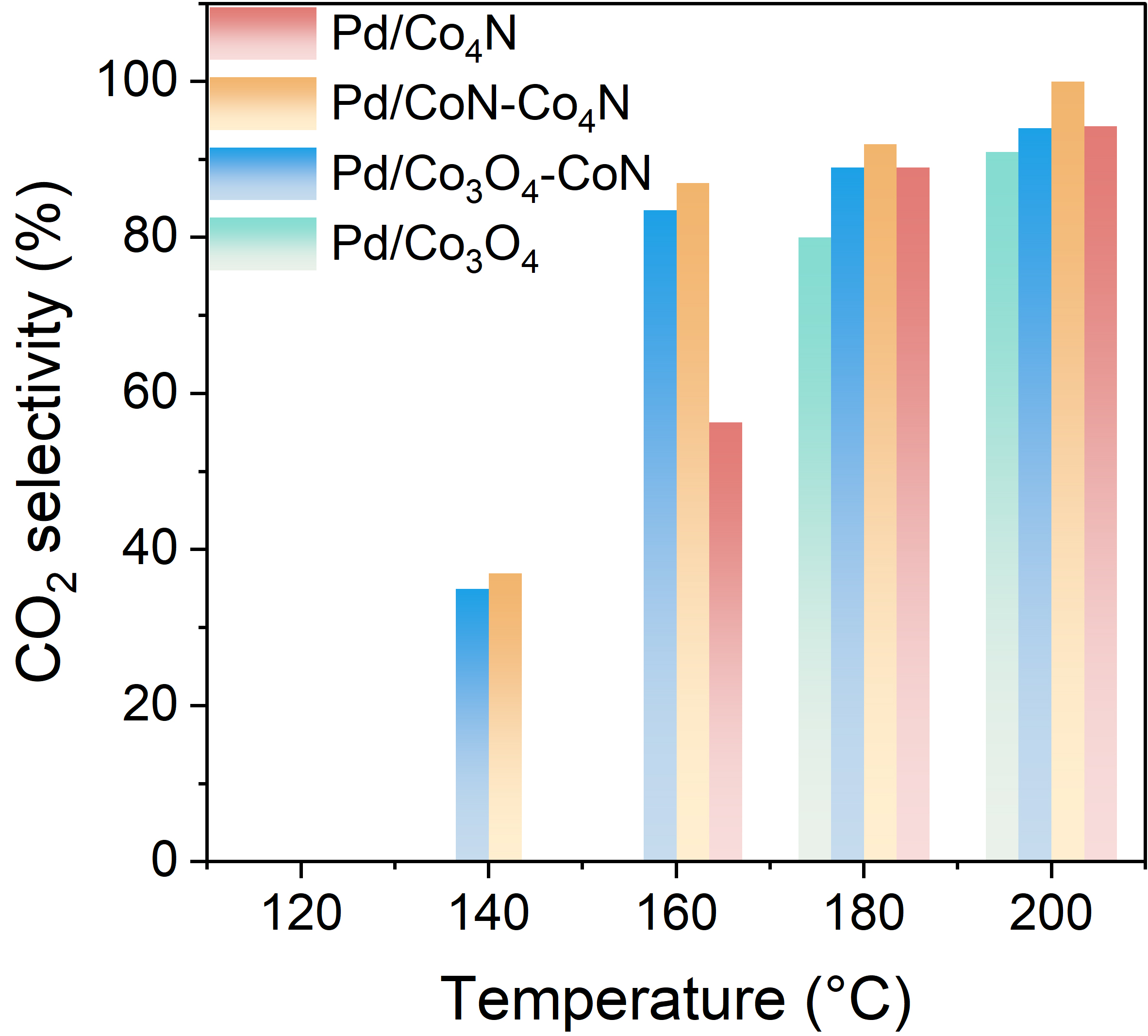
**

**Figure S14.** CO_2_ selectivity of supported-Pd catalysts for the oxidation of MEK.

**
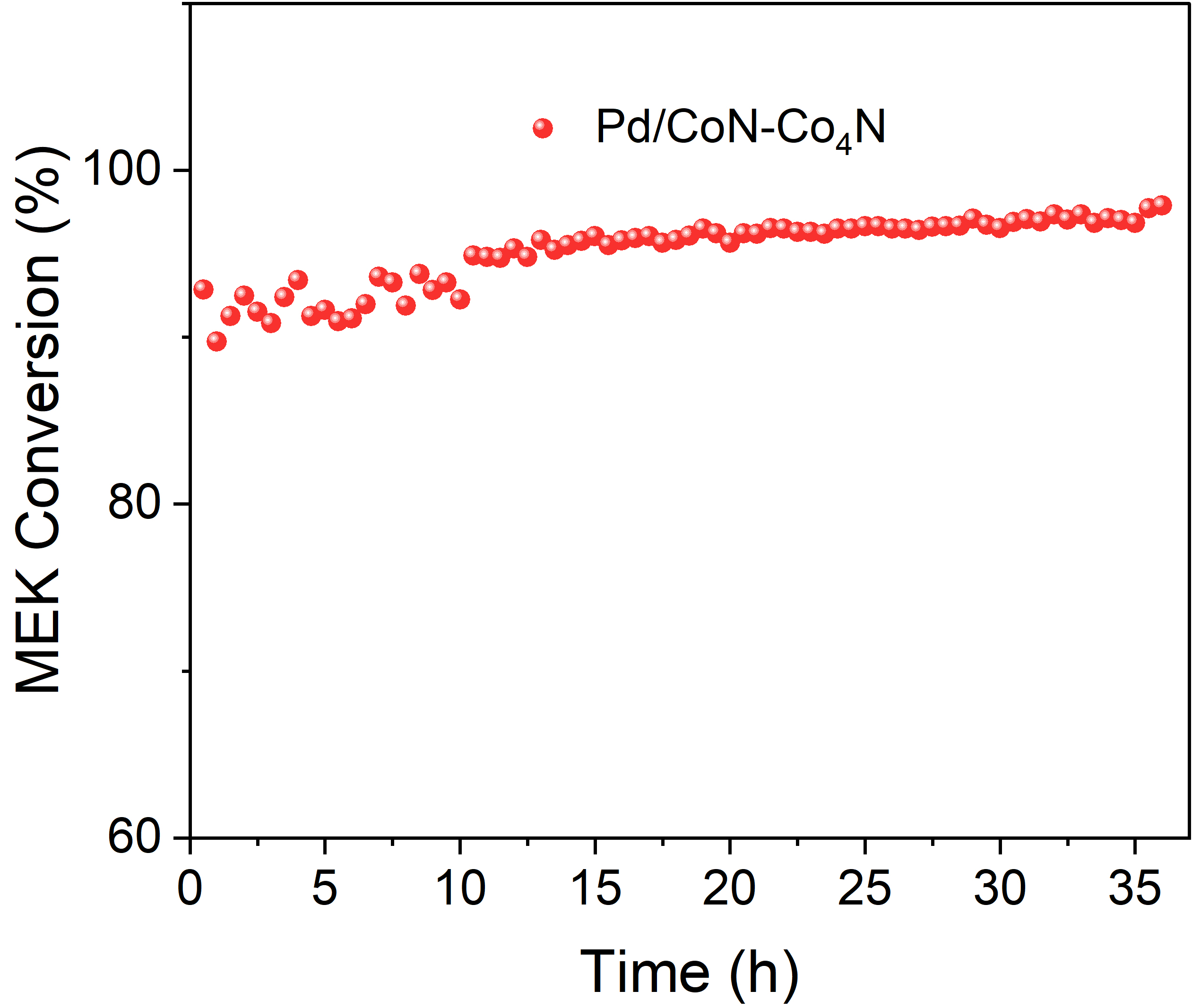
**

**Figure S15.** Long-term stability test of Pd/CoN-Co_4_N catalyst for MEK oxidation at 172 °C.

**
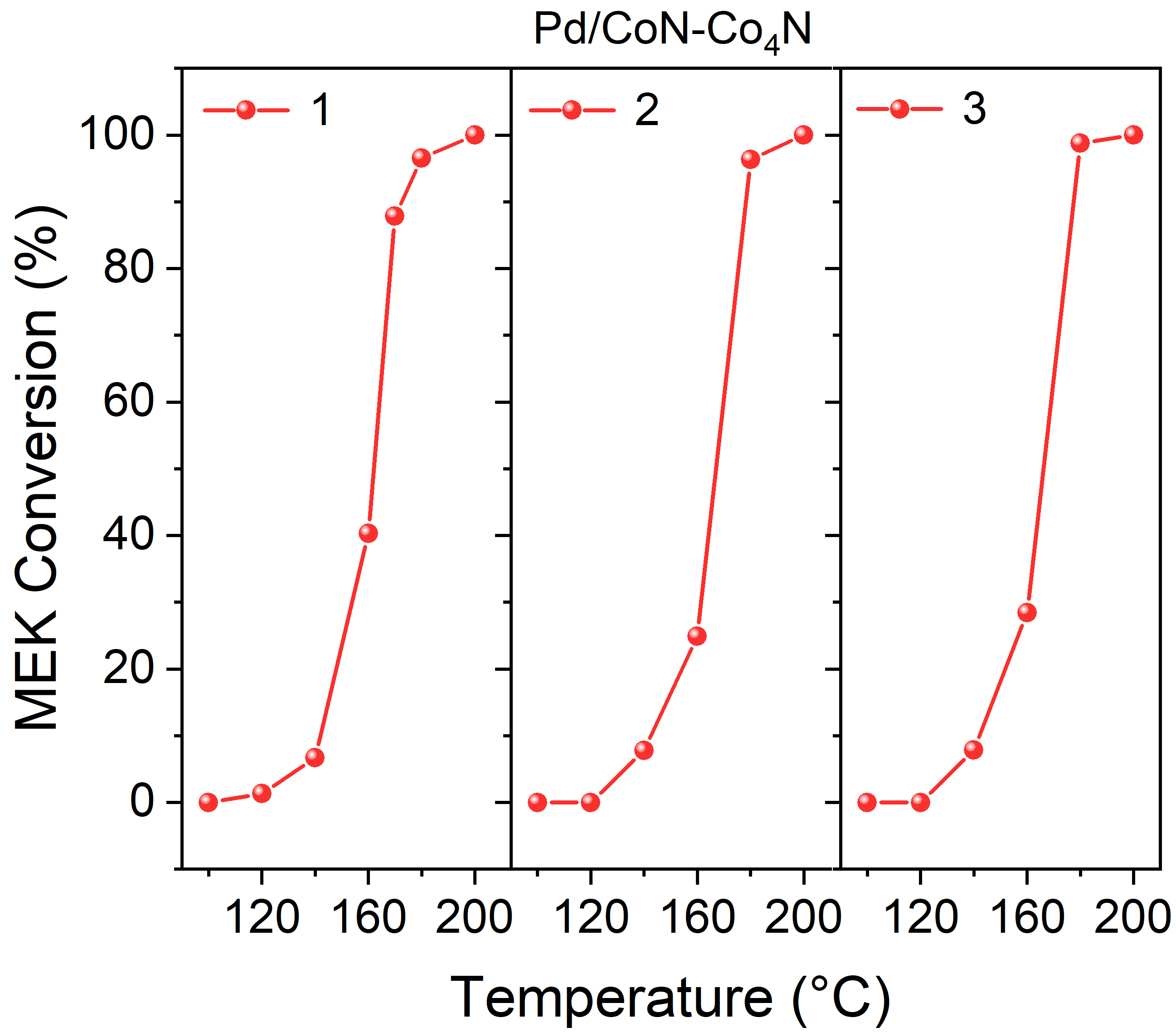
**

**Figure S16.** Cyclic stability test of Pd/CoN-Co_4_N catalyst for MEK oxidation.

**
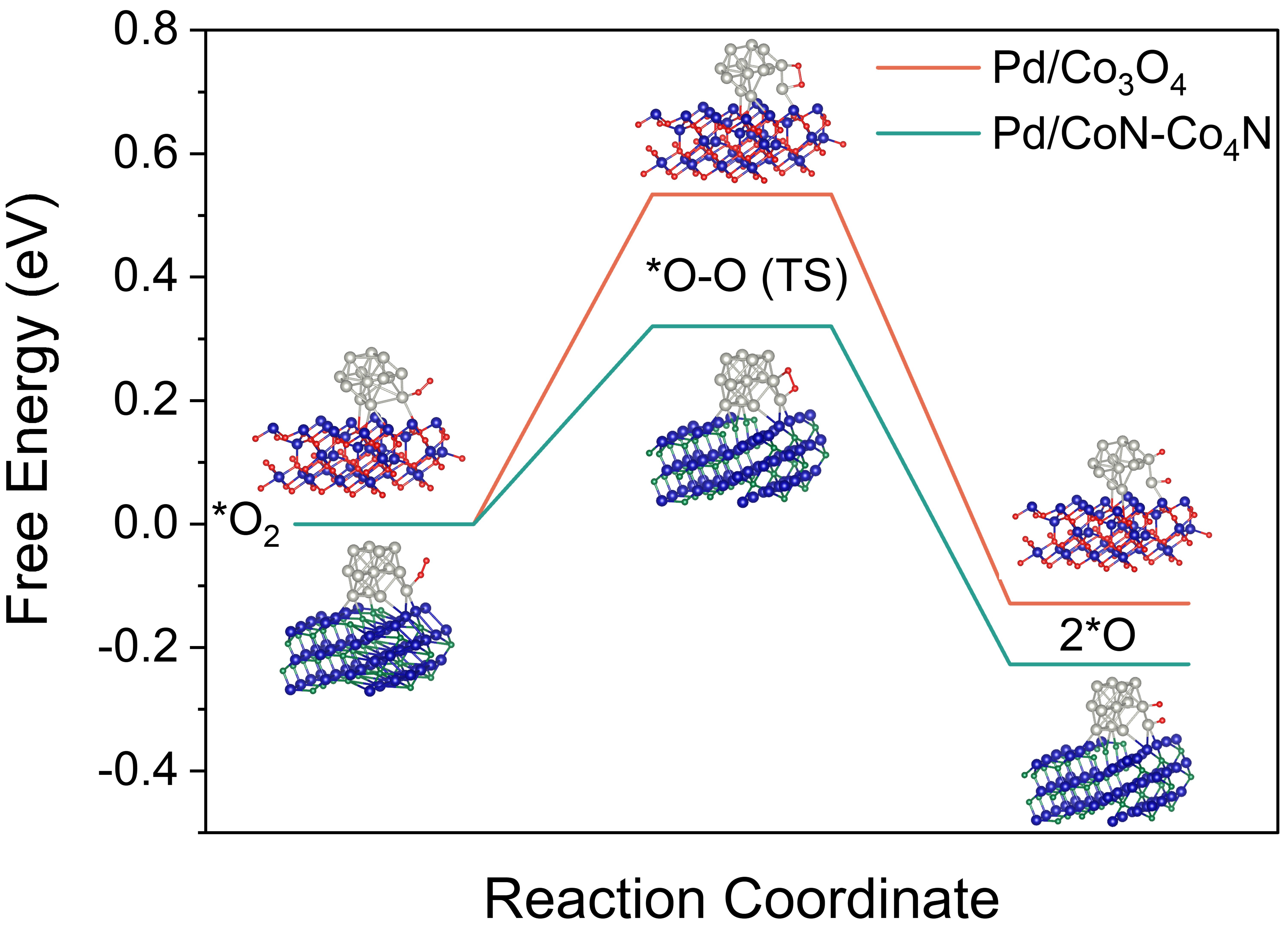
**

**Figure S17.** Calculated energy barriers for O_2_ dissociation on Pd/Co_3_O_4_ and Pd/CoN-Co_4_N (color code: Co (blue), O (red), Pd (gray), and N (green)).

**
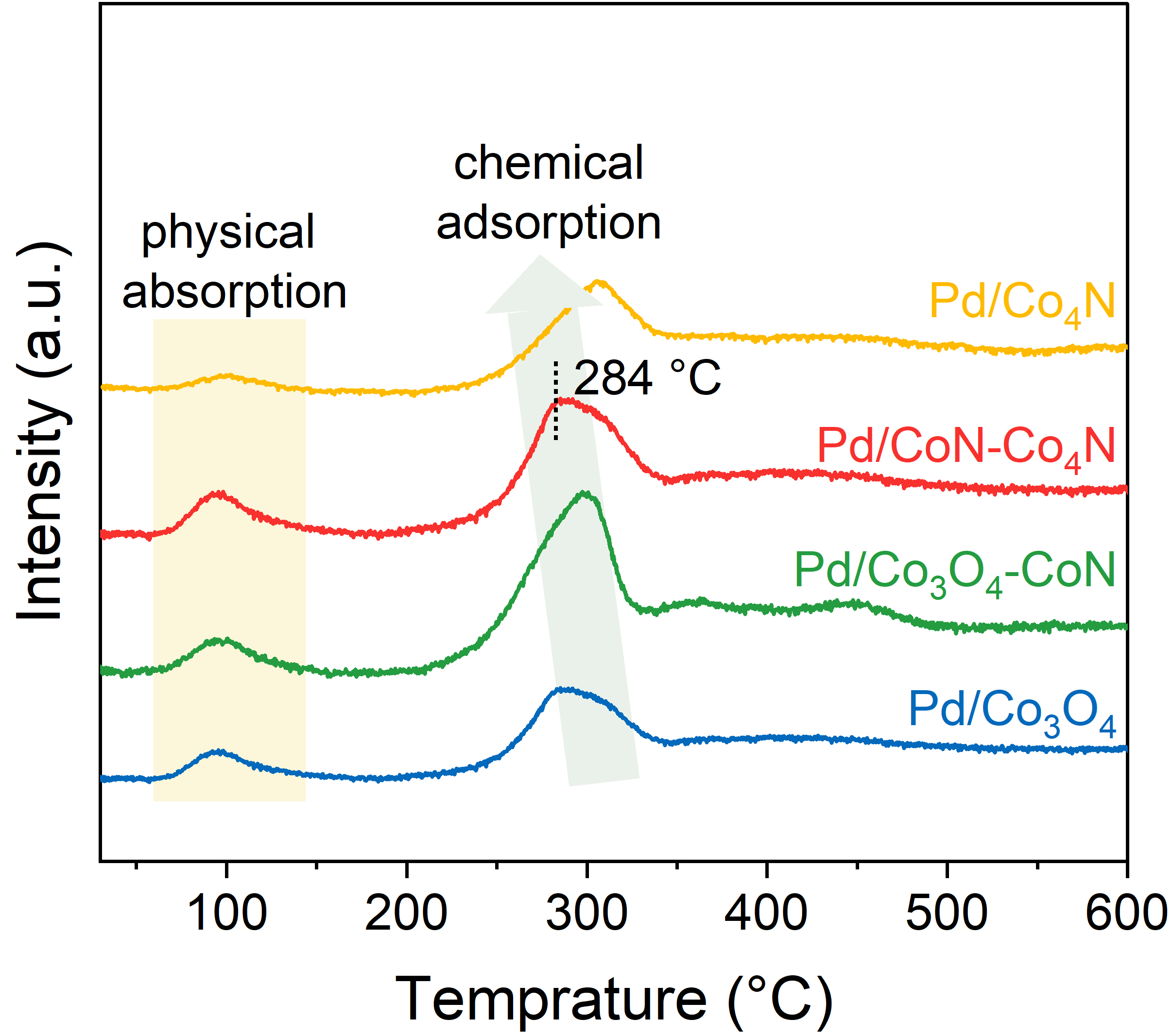
**

**Figure S18.** MEK-TPD of supported-Pd catalysts.

**
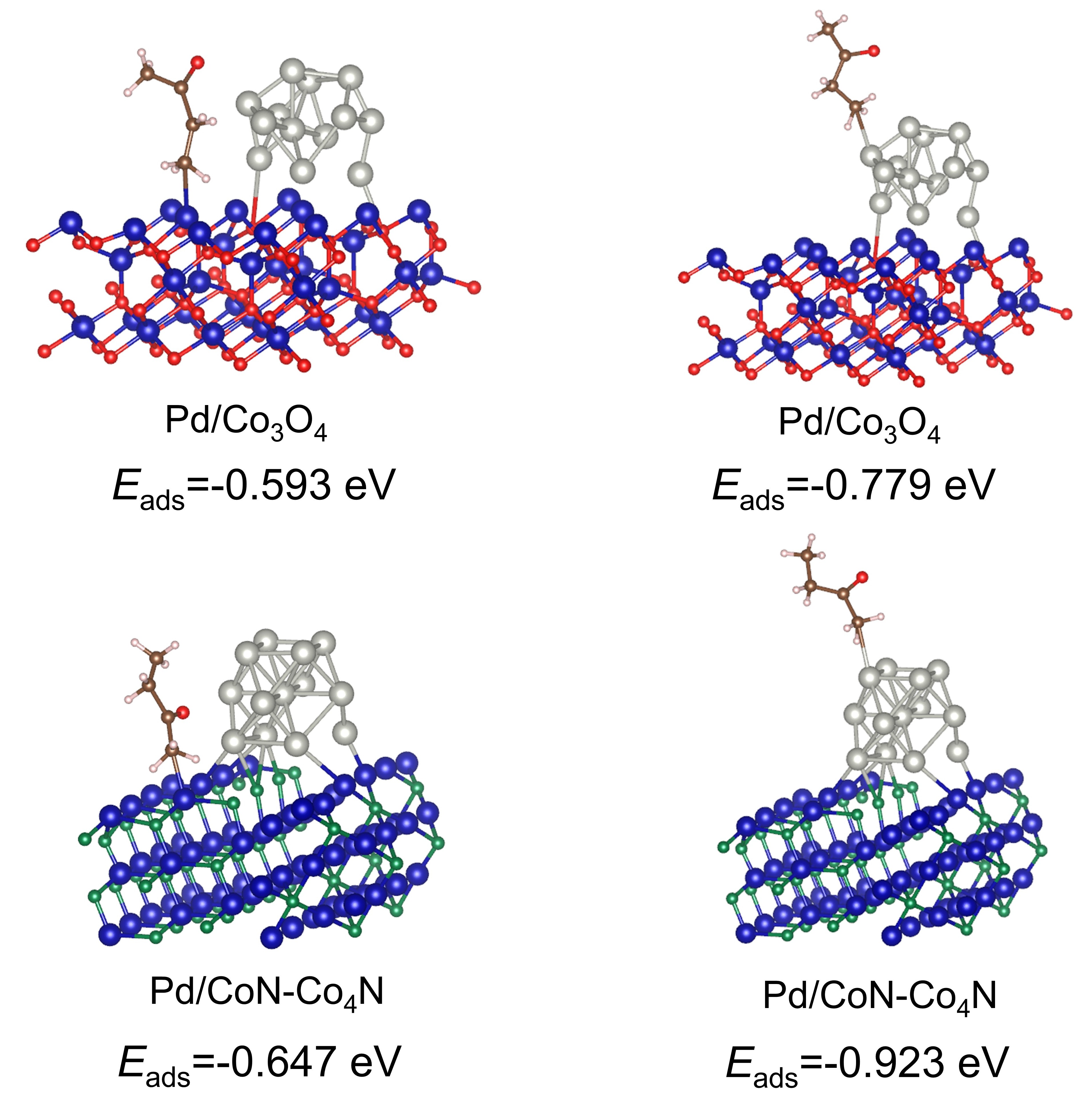
**

**Figure S19.** MEK adsorption energy on bulk Pd and Co sites of Pd/Co_3_O_4_ and Pd/CoN-Co_4_N (color code: Co (blue), O (red), Pd (gray), and N (green)).

**
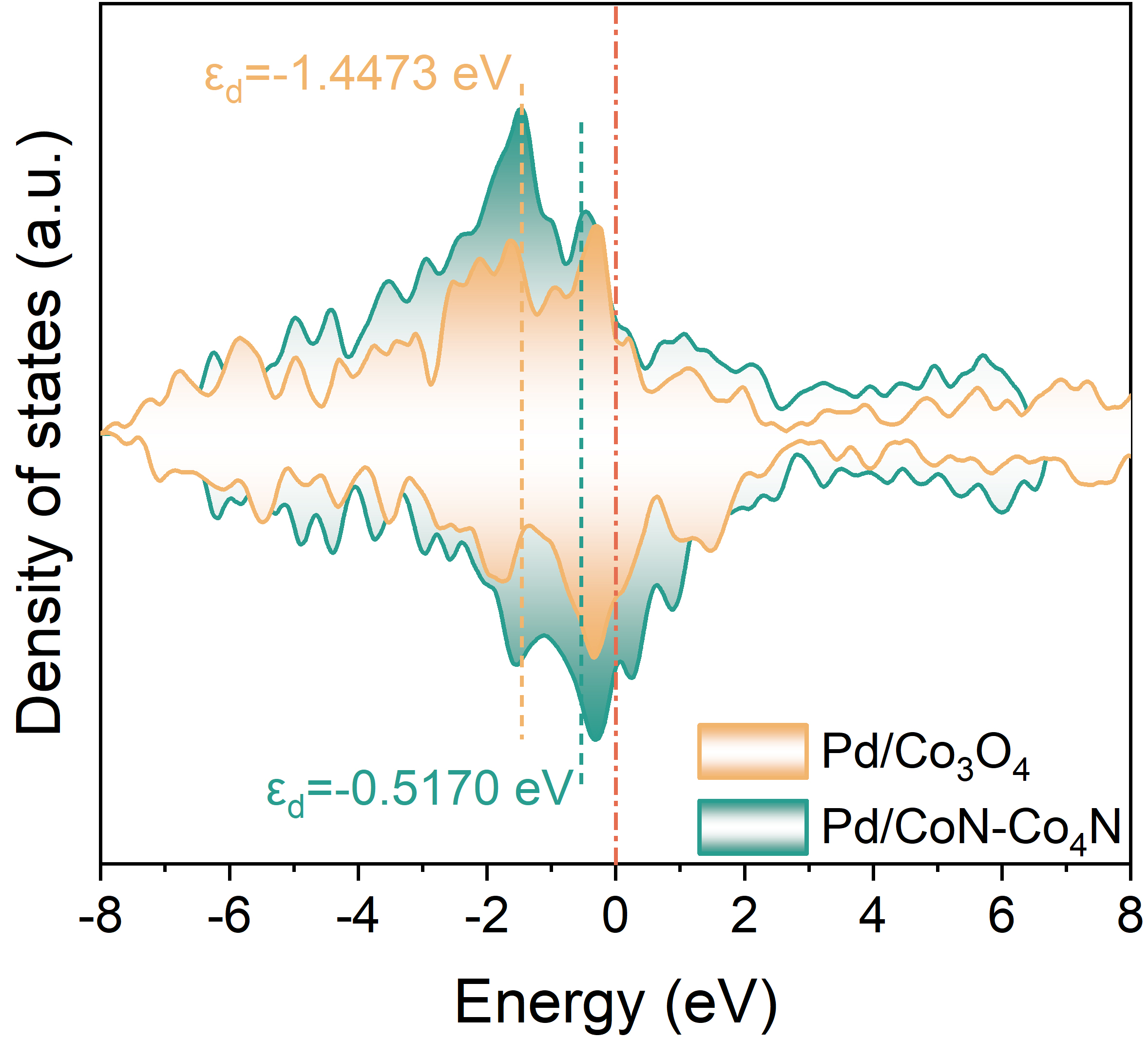
**

**Figure S20.** Calculated total density of states (TDOS) of Pd/Co_3_O_4_ and Pd/CoN-Co_4_N catalysts.

**
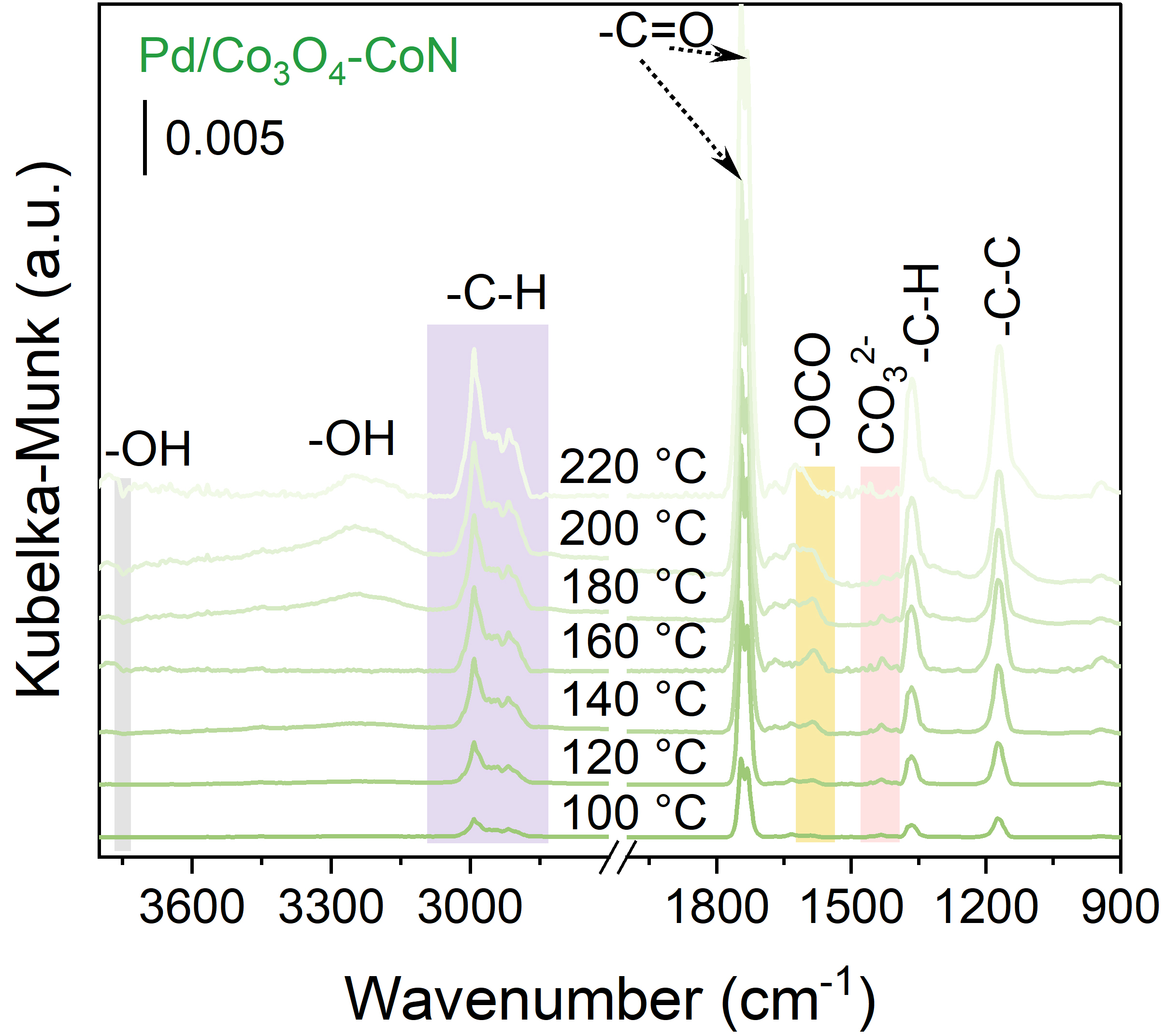
**

**Figure S21.** *In situ* DRIFTS of MEK oxidation as a function of temperature from 100 to 220 °C of Pd/Co_3_O_4_-CoN catalyst.

**
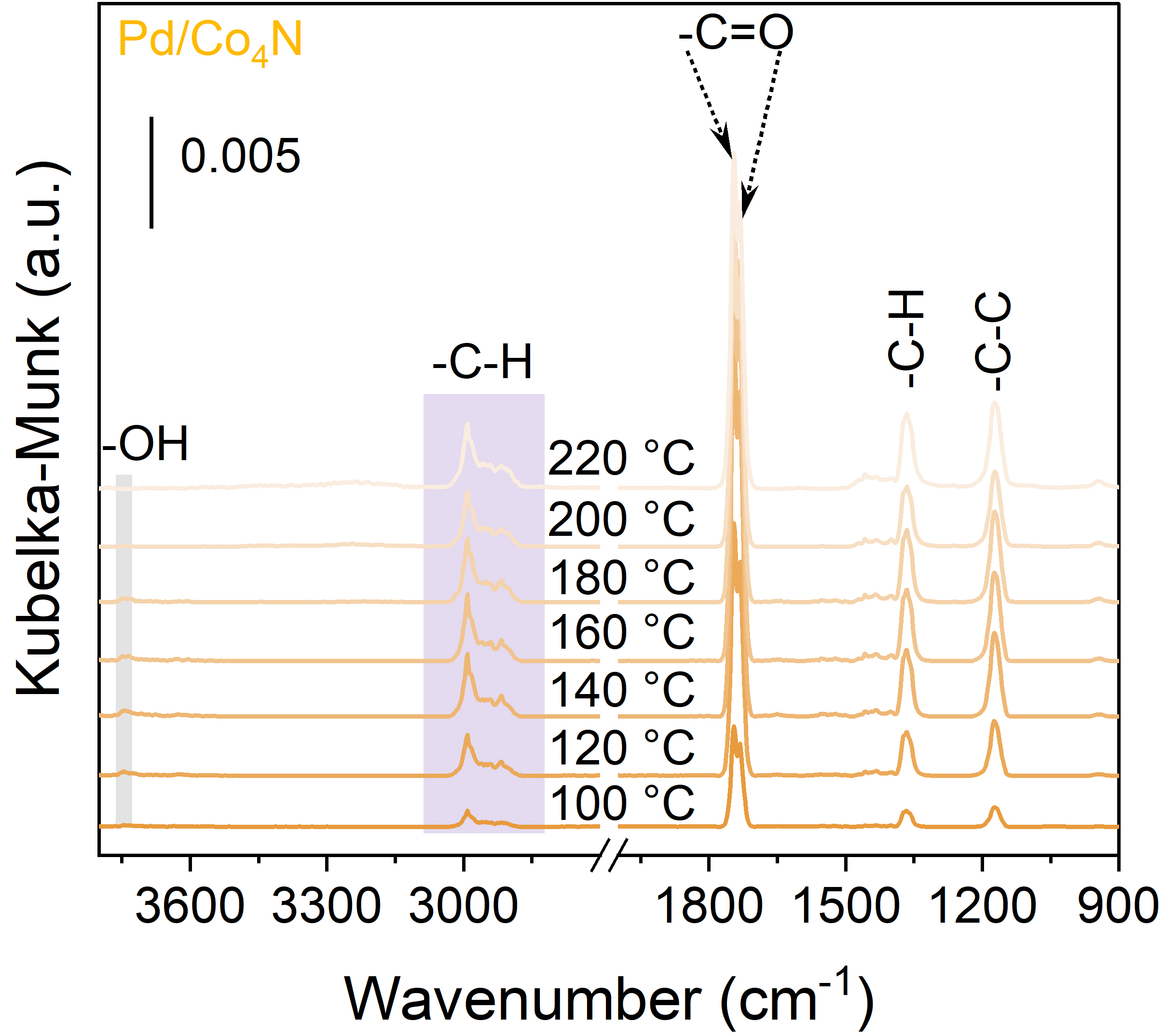
**

**Figure S22.** *In situ* DRIFTS of MEK oxidation as a function of temperature from 100 to 220 °C of Pd/Co_4_N catalyst.

**
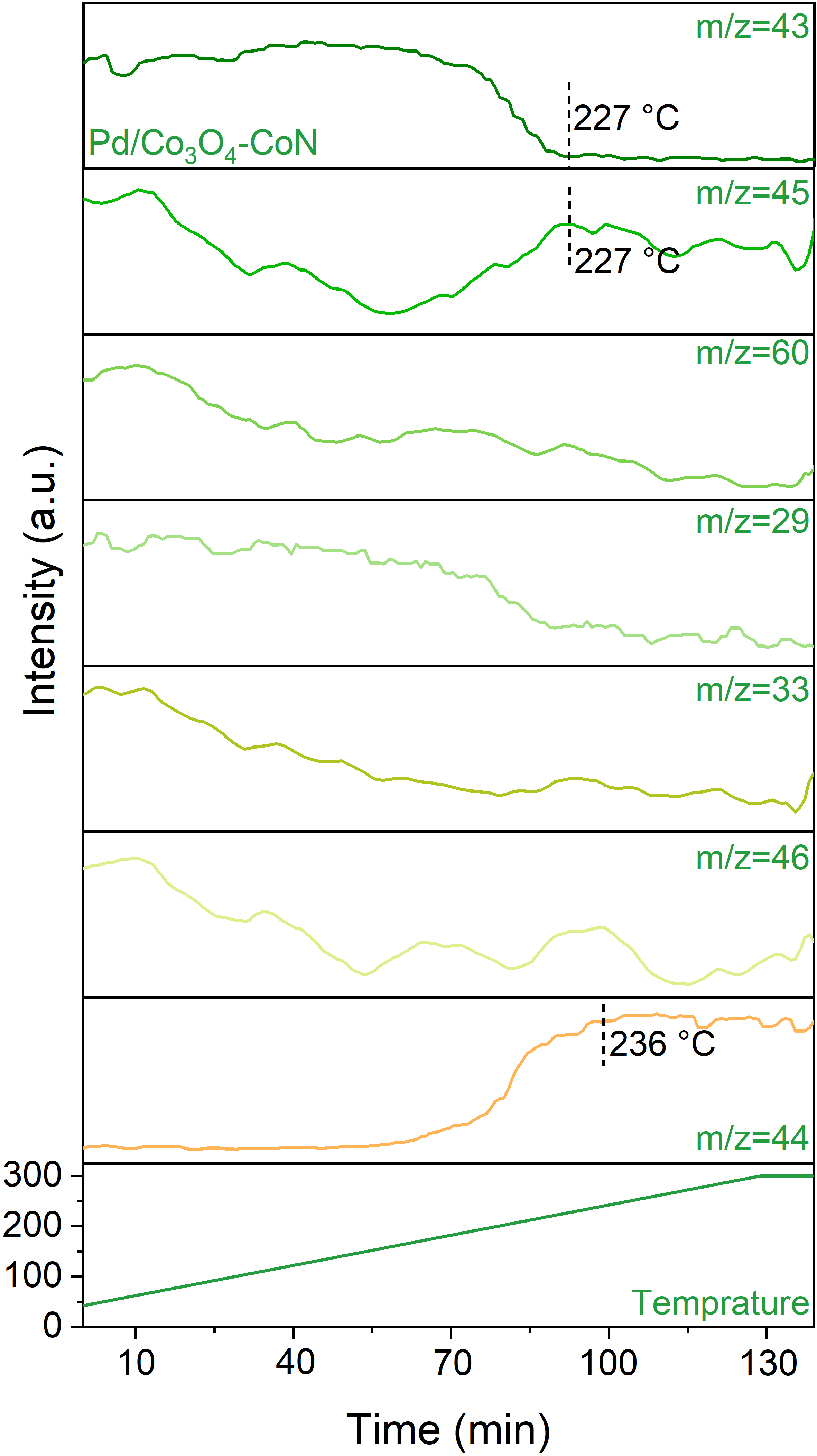
**

**Figure S23.** MEK-TPSR profiles of MEK oxidation for Pd/Co_3_O_4_-CoN catalyst.

**
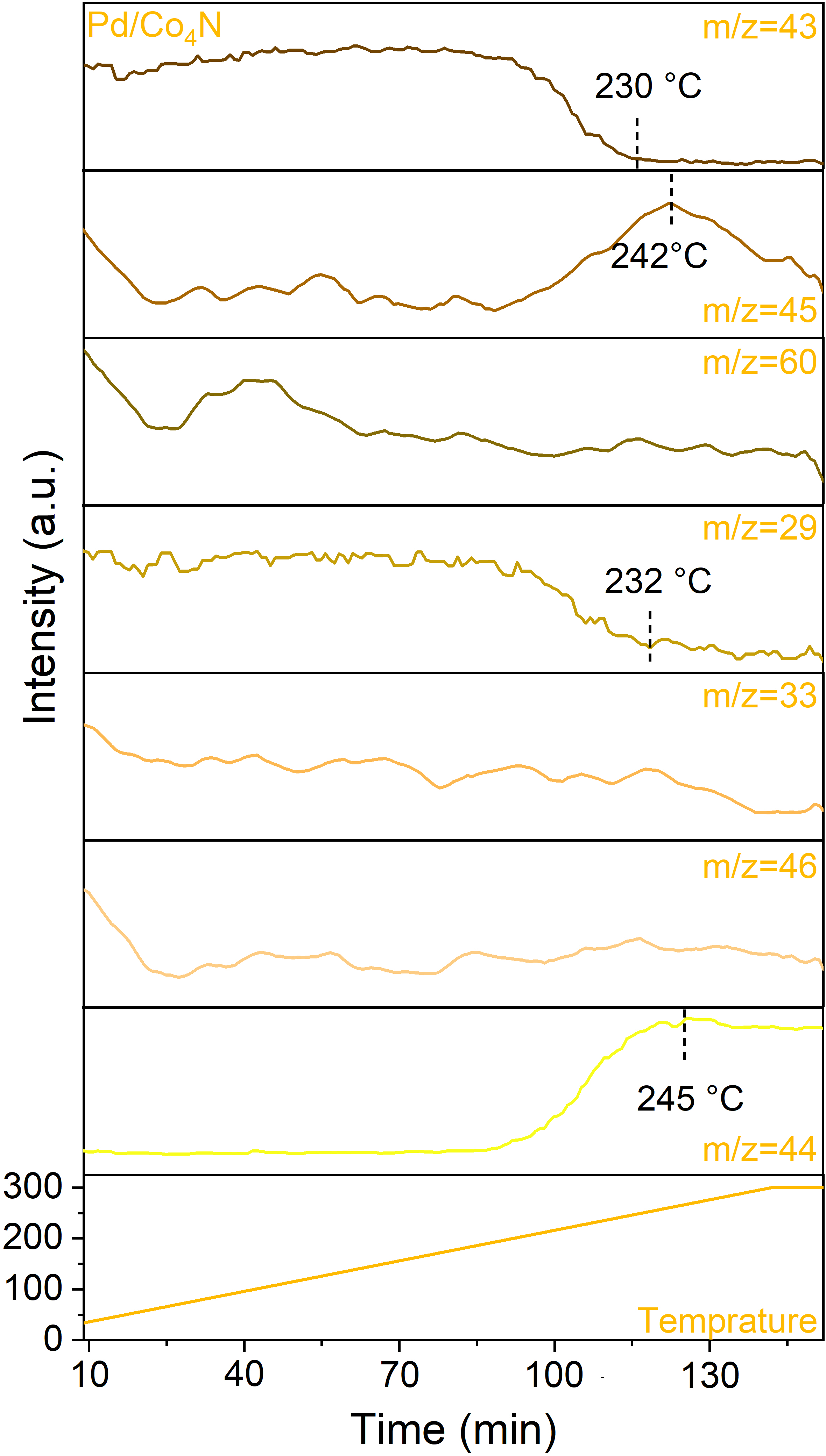
**

**Figure S24.** MEK-TPSR profiles of MEK oxidation for Pd/Co_4_N catalyst.

**
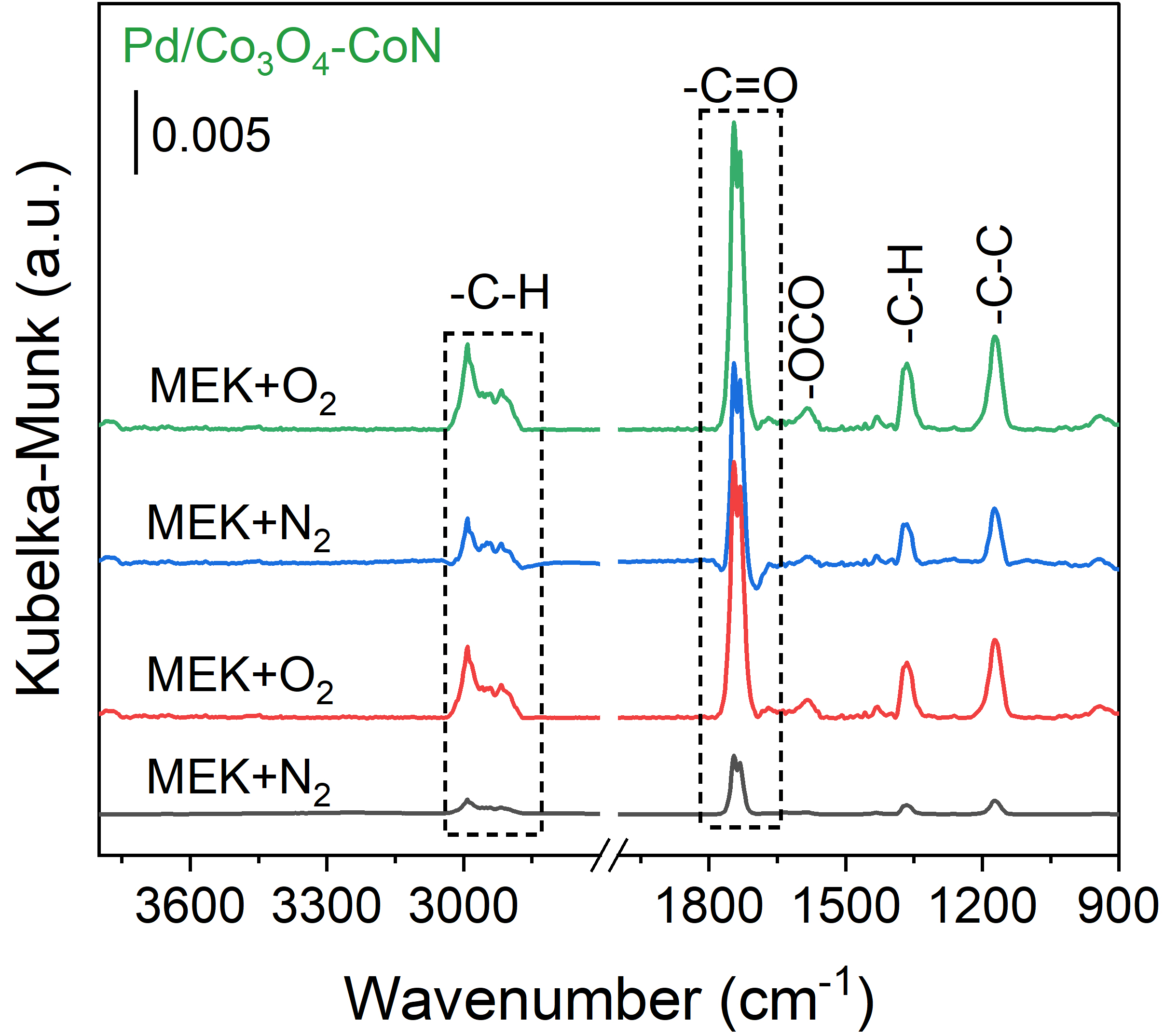
**

**Figure S25.** *In situ* DRIFTS of MEK oxidation under different reaction conditions at *T*_90_ over Pd/Co_3_O_4_-CoN catalyst.

**
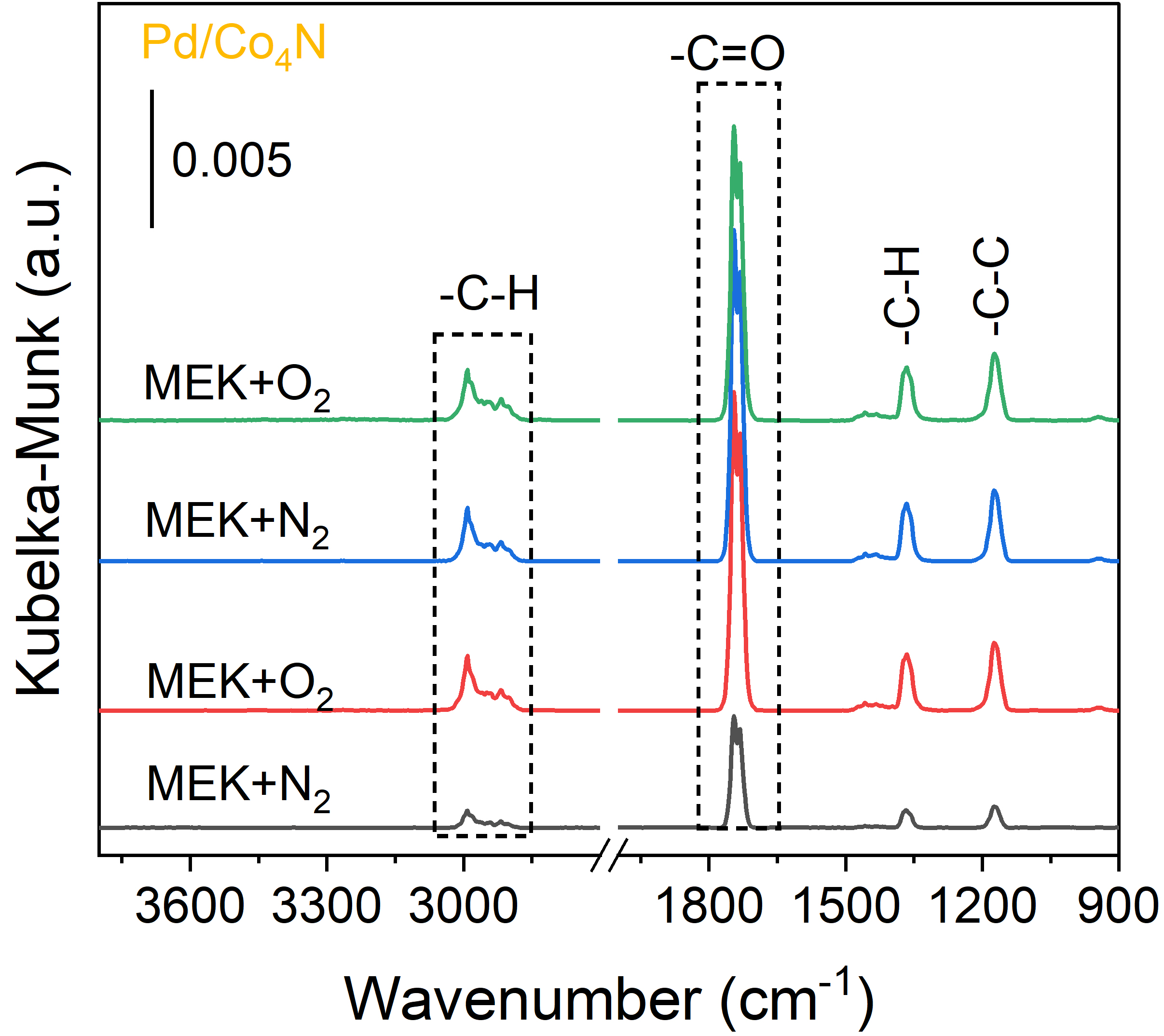
**

**Figure S26.** *In situ* DRIFTS of MEK oxidation under different reaction conditions at *T*_90_ over Pd/Co_4_N catalyst.

**
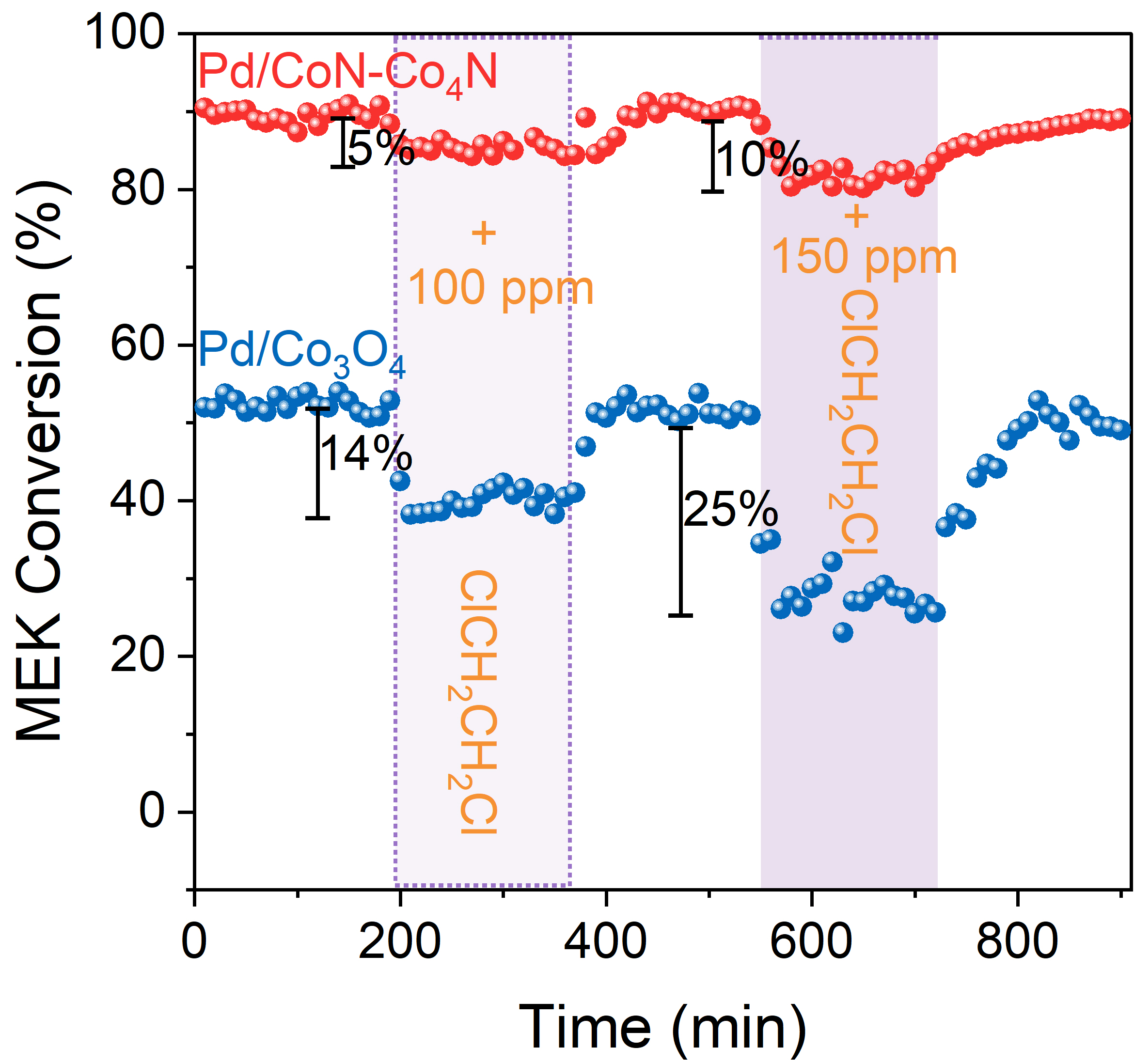
**

**Figure S27.** The stability of Pd/Co_3_O_4_ and Pd/CoN-Co_4_N catalysts for MEK oxidation under 100 and 150 ppm 1,2-dichloroethane at 172 °C.

**
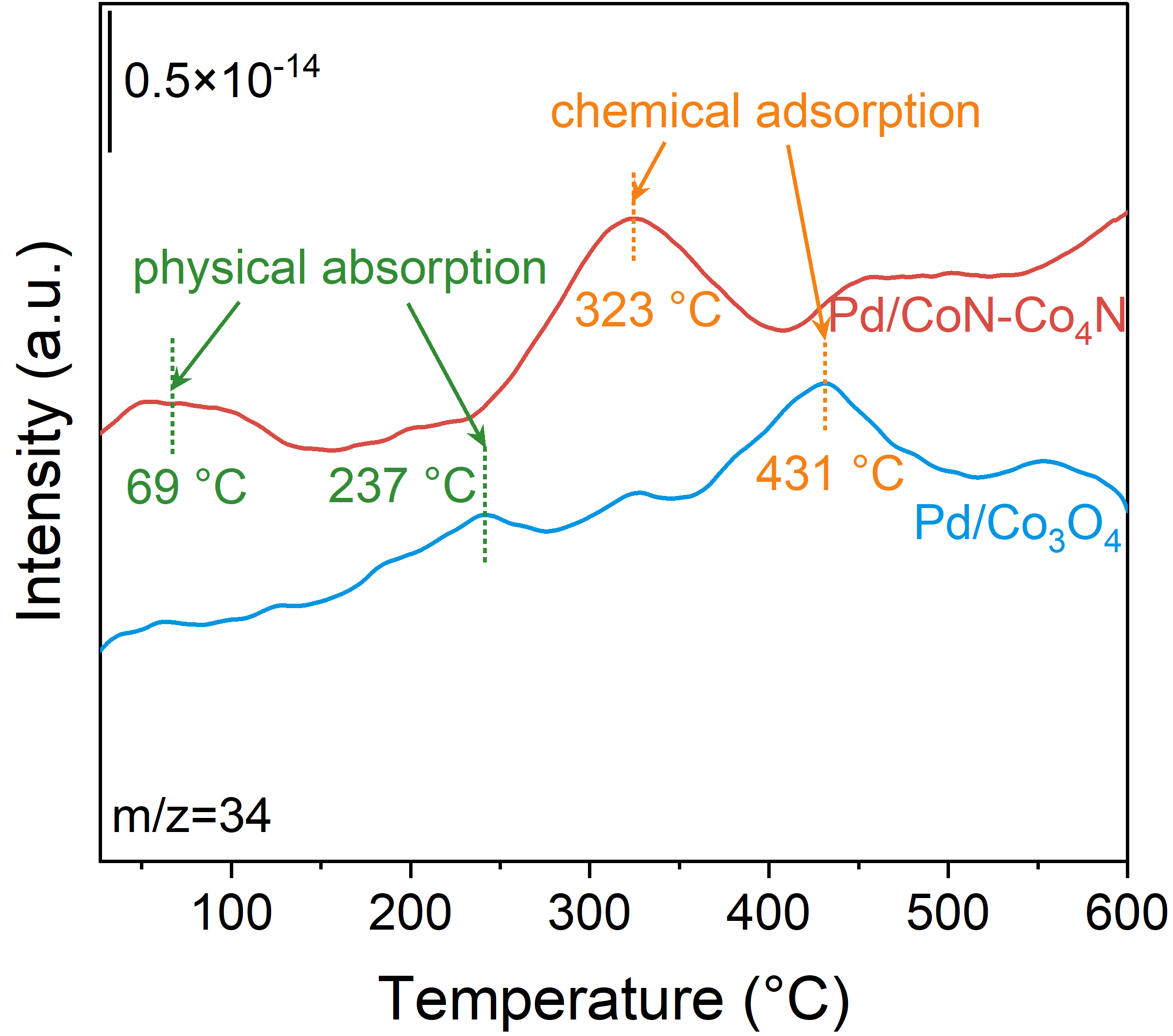
**

**Figure S28.** H_2_S-TPD-MS profiles of supported-Pd catalysts.

**
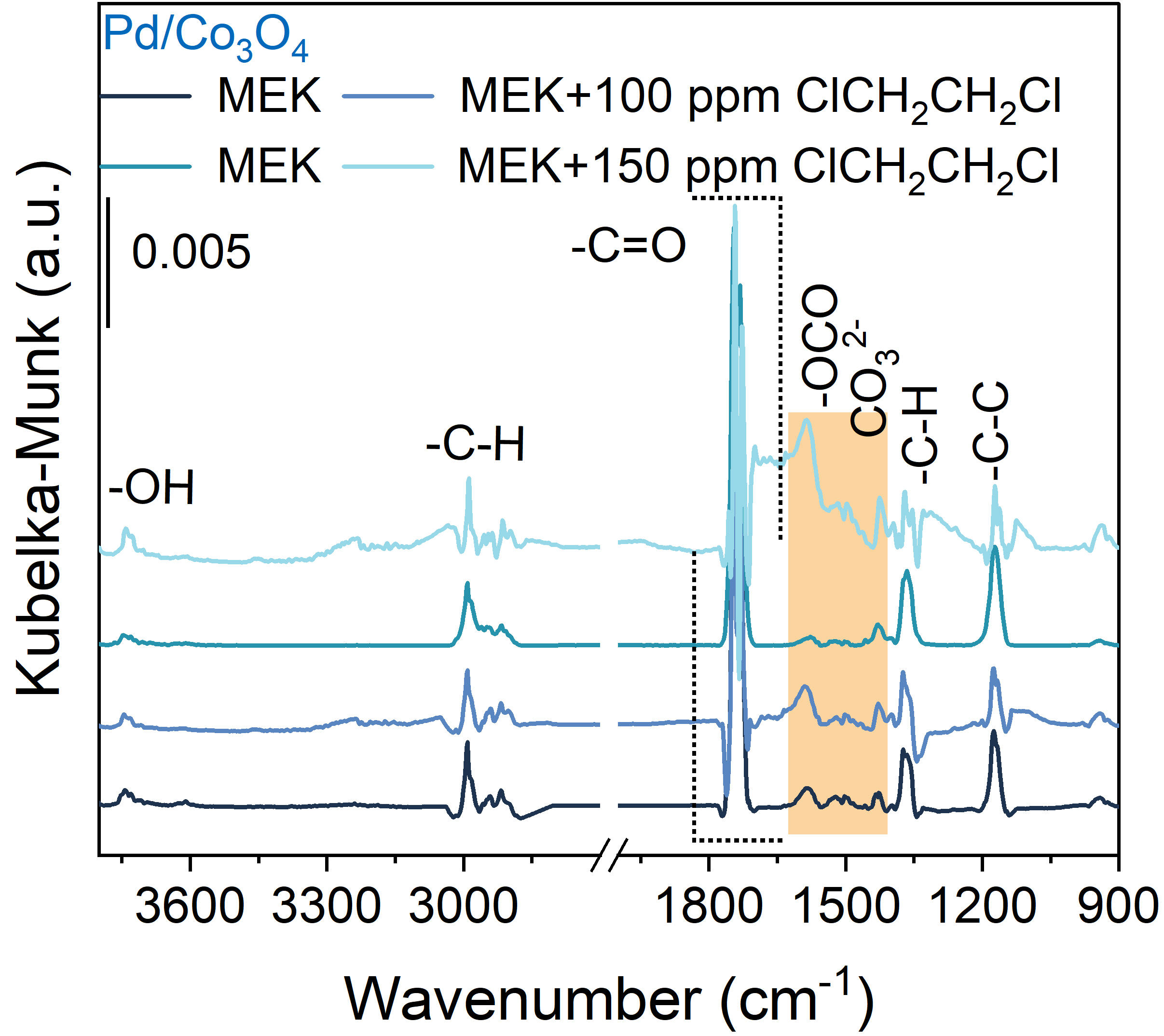
**

**Figure S29.** *In situ* DRIFTS of MEK oxidation with and without 1,2-dichloroethane over Pd/Co_3_O_4_ catalysts.

**
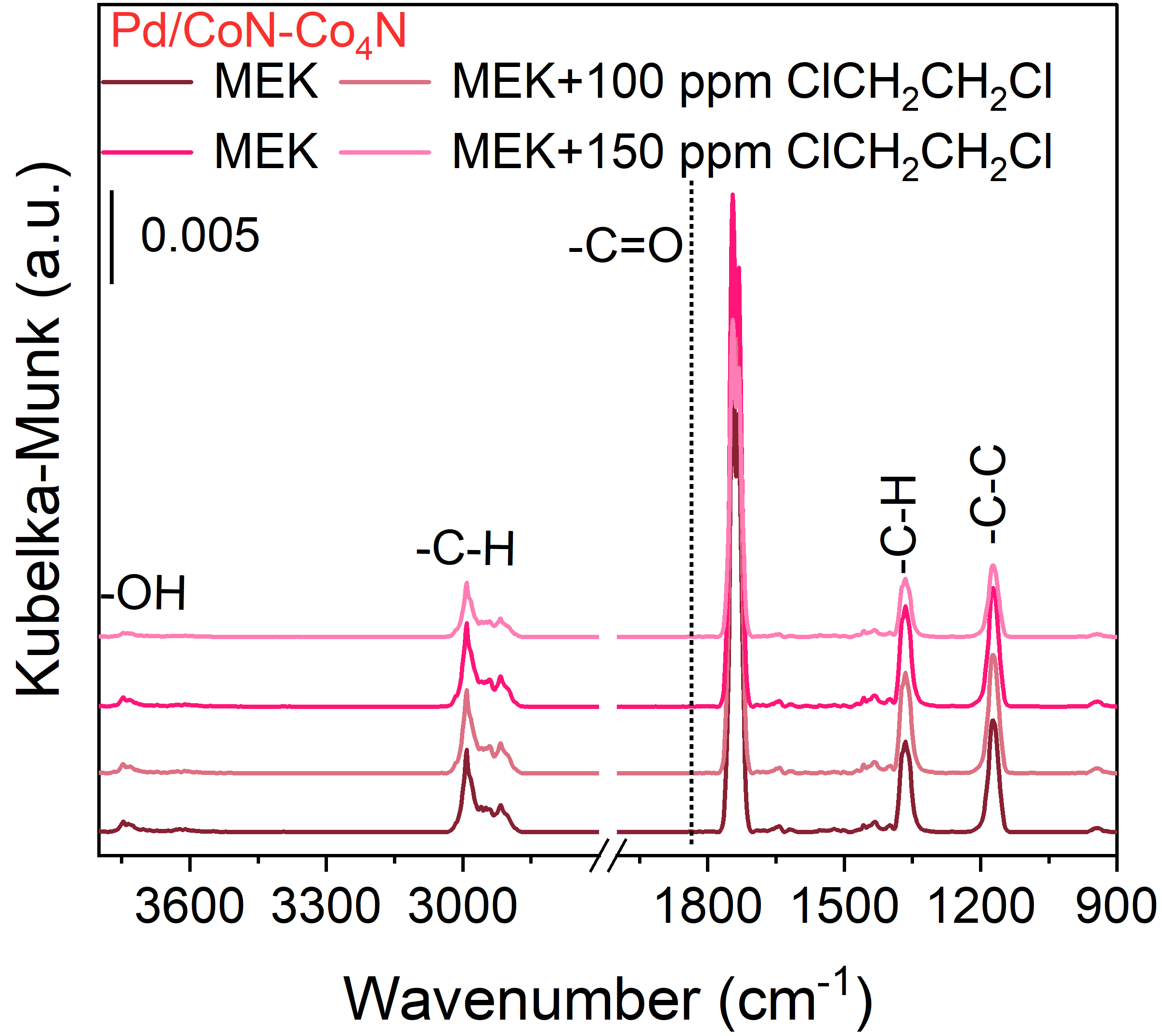
**

**Figure S30.** *In situ* DRIFTS of MEK oxidation with and without 1,2-dichloroethane over Pd/CoN-Co_4_N catalyst.

**Table S1.** Pd content and catalytic activity of supported-Pd catalysts.

| Samples | Pd content^a^  (wt.%) | Pd^0^/Pd_all_^b^  (%) | Co^2+^/Co_all_^b^  (%) | *T*_50_  (°C) | *T*_90_  (°C) |
| --- | --- | --- | --- | --- | --- |
| Pd/Co_3_O_4_ | 0.453 | 6 | 69.8 | 176 | 197 |
| Pd/Co_3_O_4_-CoN | 0.449 | 26 | 74.2 | 166 | 185 |
| Pd/CoN-Co_4_N | 0.458 | 74 | 83.9 | 161 | 172 |
| Pd/Co_4_N | 0.466 | 63 | 81.3 | 168 | 183 |

^a^ Actual Pd content obtained by ICP-OES. ^b^ Proportions of various species obtained by XPS.

**Table S2.** The MEK oxidation performance of Pd/CoN-Co_4_N compared with reported catalysts.

| Samples | *T*_50_  (°C) | *T*_90_  (°C) | Ref*.* |
| --- | --- | --- | --- |
| Pd-Ce/ZSM-5 | ~210 | ~220 | 7 |
| Mn_3_O_4_ | 182 | 194 | 8 |
| MnO*_x_* {101} | ~150 | 185 | 9 |
| Ru_25_Mn_21_/meso-TiO_2_ | 226 | 248 | 10 |
| Pt/Al_2_O_3_ | ~225 | ~260 | 11 |
| Pd/CoN-Co_4_N | 161 | 172 | This work |

**Table S3.** MEK adsorption energy on Pd and Co sites over Pd/Co_3_O_4_ and Pd/CoN-Co_4_N.

| Samples | Adsorption Site | Adsorption Configuration | Adsorption energy  (eV) |
| --- | --- | --- | --- |
| Pd/Co_3_O_4_ | interface Pd | Carbonyl O | −1.793 |
|  | interface Co | Carbonyl O | −1.015 |
|  | bulk Pd (methyl end) | C-H bond | −0.779 |
|  | bulk Co (methyl end) | C-H bond | −0.593 |
| Pd/CoN-Co_4_N | interface Pd | Carbonyl O | −1.885 |
|  | interface Co | Carbonyl O | −1.247 |
|  | bulk Pd (methyl end) | C-H bond | −0.923 |
|  | bulk Co (methyl end) | C-H bond | −0.647 |

**REFERENCES**

[1] J. P. Perdew, K.Burke, M. Ernzerhof. Generalized Gradient Approximation Made Simple. *Phys. Rev. Lett.* **1997**, *78* (7), 1396-1396, DOI: 10.1103/PhysRevLett.78.1396.

[2] G.Kresse, J. Furthmuller. Efficiency of Ab-Initio Total Energy Calculations for Metals and Semiconductors using a Plane-Wave Basis Set. *Comput. Mater. Sci.* **1996**, *6* (1), 15-50, DOI: 10.1016/0927-0256(96)00008-0.

[3] G. Kresse, J. Furthmuller. Efficient Iiterative Schemes for Ab Initio Total-Energy Calculations using a Plane-Wave Basis Set. *Phys. Rev. B* **1996**, *54* (16), 11169-11186, DOI: 10.1103/PhysRevB.54.11169.

[4] P. E. Blöchl. Projector Augmented-Wave Method. *Phys. Rev. B* **1994**, *50* (24), 17953-17979. DOI: 10.1103/PhysRevB.50.17953.

[5] G. Kresse, D. Joubert. From Ultrasoft Pseudopotentials to the Projector Augmented-Eave Method. *Phys. Rev. B* **1999**, *59* (3), 1758-1775, DOI: 10.1103/PhysRevB.59.1758.

[6] S. Grimme, J. Antony, S. Ehrlich, H. Krieg. A Consistent and Accurate Ab Initio Parametrization of Density Functional Dispersion Correction (DFT-D) for the 94 Elements H-Pu. *J. Chem. Phys.* **2010**, *132* (15), 19, DOI: 10.1063/1.3382344.

[7] L. Yue, C. He, X. Y. Zhang, P. Li, Z. Wang, H. L. Wang, Z. P. Hao. Catalytic Behavior and Reaction Routes of MEK Oxidation over Pd/ZSM-5 and Pd-Ce/ZSM-5 Catalysts. *J. Hazard. Mater.* **2013**, *244*, 613-620, DOI: 10.1016/j.jhazmat.2012.10.048.

[8] H. Pan, Y. F. Jian, C. W. Chen, C. He, Z. P. Hao, Z. X. Shen, H. X. Liu. Sphere-Shaped Mn_3_O_4_ Catalyst with Remarkable Low-Temperature Activity for Methyl-Ethyl-Ketone Combustion. *Environ. Sci. Technol.* **2017**, *51* (11), 6288-6297, DOI: 10.1021/acs.est.7b00136.

[9] Y. F. Jian, M. D. Ma, C. W. Chen, C. Liu, Y. K. Yu, Z. P. Hao, C. He. Tuning the Micromorphology and Exposed Facets of MnO*_x_* Promotes Methyl Ethyl Ketone Low-Temperature Abatement: Boosting Oxygen Activation and Electron Transmission. *Catal. Sci. Technol.* **2018**, *8* (15), 3863-3875, DOI: 10.1039/c8cy00444g.

[10] J. Wang, L. Y. Dai, J. G. Deng, Y. X. Liu, L. Jing, X. Q. Hao, W. B. Pei, X. H. Yu. Rastegarpanah, A.; Dai, H. X. An Investigation on Catalytic Performance and Reaction Mechanism of RuMn/meso-TiO_2_ Derived from RuMn Intermetallic Compounds for Methyl Ethyl Ketone Oxidation. *Appl. Catal. B-Environ.* **2021**, *296*, 120361, DOI: 10.1016/j.apcatb.2021.120361.

[11] G. Arzamendi, R. Ferrero, A. R. Pierna, L. M. Gandia. Kinetics of Methyl Ethyl Ketone Combustion in Air at Low Concentrations over a Commercial Pt/Al_2_O_3_ Catalyst. *Ind. Eng. Chem. Res.* **2007**, *46* (26), 9037-9044, DOI: 10.1021/ie071156b.
